# The Role of Platelet-Derived Microparticles in Platelet Adhesion and Aggregation to Immobilized Extracellular Matrix Proteins Under Flow

**DOI:** 10.1007/s12195-026-00920-2

**Published:** 2026-06-10

**Authors:** Tatiana Mencarini, Yana Roka-Moiia, Eleonora Puce, Anna Zucchi, Silvia Bozzi, Alberto Redaelli, Marvin J. Slepian

**Affiliations:** 1 Department of Electronics, Information and Bioengineering, Politecnico di Milano, Milano, MI Italy; 2 Department of Medicine, Sarver Heart Center, University of Arizona Health Sciences, University of Arizona, Tucson, AZ 85723 USA; 3 Department of Biomedical Engineering, University of Arizona, Tucson, AZ USA; 4 Arizona Center for Accelerated Biomedical Innovation, University of Arizona, Tucson, AZ USA

**Keywords:** Platelet adhesion, Platelet aggregation, Extracellular matrix proteins, Platelet-derived microparticles, Shear stress, Flow-based microfluidic assay, Thrombosis

## Abstract

**Introduction:**

Platelet adhesion and aggregation on exposed vascular extracellular matrix (ECM) is critical for haemostasis, with dysregulation and inappropriate thrombus formation associated with cardiovascular disease. While collagen is recognised as vital in these processes, the role of other ECM proteins is less understood. Platelet-derived microparticles (PDMPs), small vesicles released by activated platelets, similarly influence haemostasis, though their modulatory effect on ECM substrates is also unclear. We investigated platelet adhesion and aggregation on various ECM proteins–collagen, fibrinogen, fibronectin, and laminin–and examined the modulatory role of PDMPs under physiologic and pathologic flow conditions.

**Methods:**

Whole blood, alone or enriched with PDMPs, was perfused through microfluidic channels coated with ECM substrates at varying shear rates. Fluorescence imaging assessed platelet behaviour measuring surface coverage, number of thrombi, mean thrombus area and thrombus height.

**Results:**

Collagen exhibited greatest overall thrombus formation, versus the other ECM substrates, with platelet adhesion and aggregation increasing at higher shear rates. Addition of PDMPs significantly reduced thrombus area (*p* < 0.01) and height (*p* < 0.05–0.01) at all shear rates. Fibrinogen supported stable but smaller thrombi, with PDMP addition resulting in increased platelet adhesion (23% increase) at low shear. Both fibronectin and laminin demonstrated weak platelet adhesion, with thrombus formation decreasing as shear rate increased, and PDMP addition slightly increasing platelet adhesion, though without influence on thrombus dimensions.

**Conclusion:**

Collagen exerts a dominant effect in platelet adhesion and aggregation across a wide range of shear, compared to fibrinogen, fibronectin and laminin. PDMPs alter platelet adhesion and resultant thrombus formation across all ECM proteins tested, likely through competitive occupation of platelet binding sites. Our findings underscore the complex interactions between ECM proteins, PDMPs, and shear forces under flow conditions.

**Supplementary Information:**

The online version contains supplementary material available at 10.1007/s12195-026-00920-2.

## Introduction

Platelet adhesion to exposed vascular subendothelial matrix at sites of vessel injury is vital for haemostasis, for preservation of vascular integrity and prevention of blood loss. Dysregulation of this process can lead to untoward pathological arterial thrombosis leading to myocardial infarction, acute limb ischemia and stroke; as well as thrombosis and embolization associated with venous thromboembolism [1–3].

Collagen and von Willebrand factor (VWF) act synergistically in the first step of this process, with immobilised VWF contributing to the initial capture and directing of platelets to the surface of the damaged vessel, and collagen providing the substrate for establishing stable bonds for firm adhesion and the triggering of platelet activation [4–6]. Adherent activated platelets release soluble agonists, e.g. adenosine diphosphate, thromboxane A2, thrombin, further recruiting circulating platelets, which upon activation gain the ability to bind to each other, leading to aggregation. The activation of platelet integrin αIIbβ3 causes a conformational change of the platelet cytoskeleton fostering stable aggregation between platelets. A concomitant signalling wave permits clot retraction, consolidating the thrombus [7–9].

The dynamics of platelet adhesion under flow is critical for thrombus formation and the overall haemostatic response [10]. Recent advances in microfluidic technology have enabled extensive studies of platelet adhesion and aggregation under flow conditions that replicate the physiological and pathological shear forces within blood vessels. However, most research has primarily focused on collagen, the most abundant protein at sites of vascular injury, and VWF, the main contributor to platelet adhesion under elevated shear stress [11–14]. Relatively few studies have explored the roles of other key extracellular matrix (ECM) proteins, i.e. fibrinogen, fibronectin, and laminin, in platelet thrombus formation [15]. Their exact contribution remains incompletely understood and continues to be a topic of ongoing research [16].

In addition to exploring the role of ECM proteins in thrombus formation, research attention has increasingly been directed towards other factors that may influence thrombus formation, notably platelet-derived microparticles (PDMPs). While some studies exist, the exact mechanism via which they contribute to or alter platelet adhesion, aggregation and thrombus formation at sites of vascular injury remains unclear. PDMPs are membrane-bound phospholipid microvesicles shed by platelets upon activation, driven by a range of initiators including ongoing coagulation, focal and systemic inflammation, hypoxia and pathologic shear stress [17]. PDMPs are the most abundant type of circulating microvesicle, accounting for 70–90% of those circulating [18]. Their contents are extremely diverse, comprising lipids, proteins, nucleic acids, and organelles depending on the mechanism that induced their release from the parent cell [19][20–22] and can regulate a wide range of biological activities [23, 24]. PDMPs are influential mediators of intercellular communication and, unlike platelets, can overcome tissue barriers, allowing platelet-derived contents to be transferred to target cells and organs that platelets cannot reach directly [25–28]. Table 1 summarises functions exerted by the biological contents of PDMPs and their association with several pathological states.

**Table 1:** Platelet-derived microparticles and their biological content and activity. Correlation with pathological states

Biological content	Biological activity	Correlation with diseases	References
Proteins: GPVI, GPIb/IX/V, αIIbβ3, P2Y12, p-selectin, thrombospondin-1, fibrinogen	Facilitate platelet adhesion, aggregation, and thrombin generation; regulate platelet activation; support clot stabilization	Thrombosis (deep vein thrombosis, pulmonary embolism), atherosclerosis (plaque formation, instability), bleeding disorders (Bernard-Soulier syndrome, Glanzmann thrombasthenia)	[29–32]
Lipid components: phosphatidylserine, sphingomyelin, cholesterol, phosphatidyletanolamine	Provide procoagulant surfaces for clotting factors; promote thrombin generation	Cardiovascular diseases, diabetes (increased microparticle levels linked to vascular complications)	[17, 20, 29]
RNA cargo: miRNAs (e.g., miR-223, miR-126, miR-21, miR-19b)	Modulate target gene expression in recipient endothelial, immune, or cancer cells; anti-apoptotic effects	Cancer (angiogenesis, metastasis), cardiovascular diseases (endothelial repair, plaque stabilization)	[33–35]
Cytokines/chemokines: IL-1β, RANTES, platelet factor 4 (PF4), VEGF, TGF-β	Stimulate inflammation, recruit immune cells, promote angiogenesis or fibrosis	Rheumatoid arthritis, inflammatory bowel disease, cancer (tumour microenvironment)	[36–39]
Adhesion molecules: ICAM-1, VCAM-1, PECAM-1	Promote interaction with leukocytes, endothelium, and other cells	Chronic inflammatory diseases, sepsis, vascular dysfunction	[35, 40–42]
Enzymes: matrix metalloproteinases, caspases	Degrade extracellular matrix, facilitate cell migration, and modulate apoptosis	Cancer metastasis, tissue injury repair	[34, 43]
Reactive oxygen species (ROS)	Induce oxidative stress, endothelial dysfunction, and mitochondrial damage	Hypertension, metabolic syndrome, aging-related vascular dysfunction	[44, 45]
Growth factors: platelet-derived growth factor (PDGF), VEGF, hepatocyte growth factor (HGF)	Stimulate endothelial repair, angiogenesis, and tissue regeneration	Wound healing, diabetic ulcers, cancer progression	[34, 46, 47]
Mitochondrial DNA	Induce inflammatory responses; act as damage-associated molecular patterns	Autoimmune diseases (e.g., lupus, multiple sclerosis), sepsis	[48, 49]

Legenda: GP, glycoprotein; IL, interleukin; RANTES, chemokine ligand 5; VEGF, vascular endothelial growth factor; TGF, transforming growth factor; ICAM, intercellular adhesion molecule; VCAM, vascular cell adhesion molecule; PECAM, platelet-endothelial cell adhesion molecule.

Interestingly, PDMPs have also been found in blood of healthy donors [50], with their levels correlating with a range of disease states [50–53], suggesting their dual anticoagulant/procoagulant function. Several studies have shown that platelet-derived microparticles likely exert regulatory effects on platelet adhesion under flow, as well as on successive steps such as platelet activation, aggregation and the trigger of the coagulation cascade [54–56]. While it is well established that different agonists stimulate the release of distinct PDMP subpopulations, each varying in number, molecular content, and surface receptor profile, in this study, we examined PDMPs generated mechanically through platelet sonication. Functionally, these PDMPs were found to be strongly procoagulant, owing to the abundant exposure of phosphatidylserine that provided a catalytic surface for thrombin generation. At the same time, they exerted a modest anti-aggregatory effect, as they partially inhibited collagen- and ADP-induced platelet aggregation, though less potently than shear-derived PDMPs [19]. Understanding the influence of PDMPs on platelet adhesion to immobilized ECM proteins and subsequent aggregation under flow conditions is crucial, as it may provide insights into the intricate interplay between platelets and their microenvironment during thrombus formation.

In the present study we hypothesized that: 1. subendothelial matrix proteins, specifically collagen, fibronectin, fibrinogen and laminin, have varied effects on platelet adhesion over a range of flow (shear stress) conditions, and 2. the addition of exogenous platelet-derived microparticles will alter the quantity and quality of platelet aggregates and thrombi formed over the same range of flow conditions. To address this, we employed a microfluidic channel system to simulate physiological blood flow and monitored the behaviour of fluorescently-labelled platelets interacting with immobilized ECM proteins, in whole blood, in the absence or presence of PDMPs.

## Methods

### Blood Collection and Preparation of Platelet-Derived Microparticles

Fresh human blood was obtained from healthy, aspirin free volunteers via venipuncture. All volunteers provided informed consent with the study conducted under protocol #1810013264 (“Optimization Cardiovascular & Mechanical Circulatory Support (MCS) Devices Thrombogenicity for Eliminating Anticoagulation”) approved by the Institutional Review Board of the University of Arizona.

For platelet adhesion studies, 8 mL of whole blood (WB) was immediately anticoagulated following venipuncture with 450 ATU/mL hirudin, stained with 1 μM 3,3′-dihexyloxacarbocyanine iodide (DiOC_6_, Sigma-Aldrich) for 15 minutes, and stored at room temperature in the dark until used within 2 hours. Alternatively, 22 mL of whole blood was anticoagulated with acid citrate dextrose solution (9:1) and centrifuged at 300 g for 15 minutes to separate platelet-rich plasma. Platelets were isolated from platelet-rich plasma by gel chromatography using Sepharose-2B and eluted in modified Tyrode’s buffer (10 mM HEPES, pH 7.4, containing 125 mM NaCl, 1 mM trisodium citrate, 2.7 mM KCl, 0.5 mM Na_2_HPO_4_, 2 mM MgCl_2_, 25 mM glucose, and 0.1% bovine serum albumin (BSA)). To generate PDMPs, 350 μL of recalcified gel-filtered platelets (100,000 platelets/μL, 2.5 mM CaCl_2_) were subjected to sonication (50% of power, 10 s, Branson Ultrasonics™ SLPt Sonifier). Sonicated samples were centrifuged (2000 g for 10 minutes x2) at room temperature to sediment platelets [19]. The supernatant containing PDMPs was collected and stored on ice for immediate use as a PDMP-rich fraction or snap-frozen at − 80 °C for later use.

### Surface Expression of Receptors on Platelets and Microparticles

Flow cytometric analysis of platelet surface receptors was carried out according to established protocols [57, 58]. Sonicated GFP samples (20000 platelets/µL, 2.5 mM CaCl₂) obtained from 8 healthy donors were stained using fluorescein-conjugated antibodies: anti-CD41–APC (Thermo Fisher Scientific); anti-GPVI–PE (BD Biosciences); anti-CD42a–FITC (Thermo Fisher Scientific); anti-CD62P–APC (Thermo Fisher Scientific); anti-CD31–FITC (Thermo Fisher Scientific).

Following staining (30 minutes), samples were fixed in 2% paraformaldehyde (Santa Cruz Biotechnology) for 20 minutes and diluted to a final volume of 1 mL with phosphate-buffered saline (Gibco™, Thermo Fisher Scientific). Flow cytometric acquisition was performed on a FACSCanto II instrument (BD Biosciences), recording 10000 events within the defined gate “Platelets + Microparticles.” Data were analysed using FCS Express V3 software (DeNovo Software).

Single platelets were distinguished from microparticles based on their forward and side scatter profiles, using standard SPHERO™ polystyrene fluorescent nanobeads (Spherotech; 880 and 1350 nm) as size references [59]. Marker-positive platelet and microparticle populations were expressed as the percentage of all events within the “Platelets + Microparticles” gate. Particle size was assessed as the median forward scatter (MFS) [60], while receptor density was expressed as the ratio of median fluorescence intensity to median forward scatter (MFI/MFS). Density of the expression of PDMPs’ adhesion receptors was normalised over the expression of resting platelets’ receptors, computed as ratio.

### Microfluidic Platform Fabrication and Protein Immobilization

**Fig. 1:**
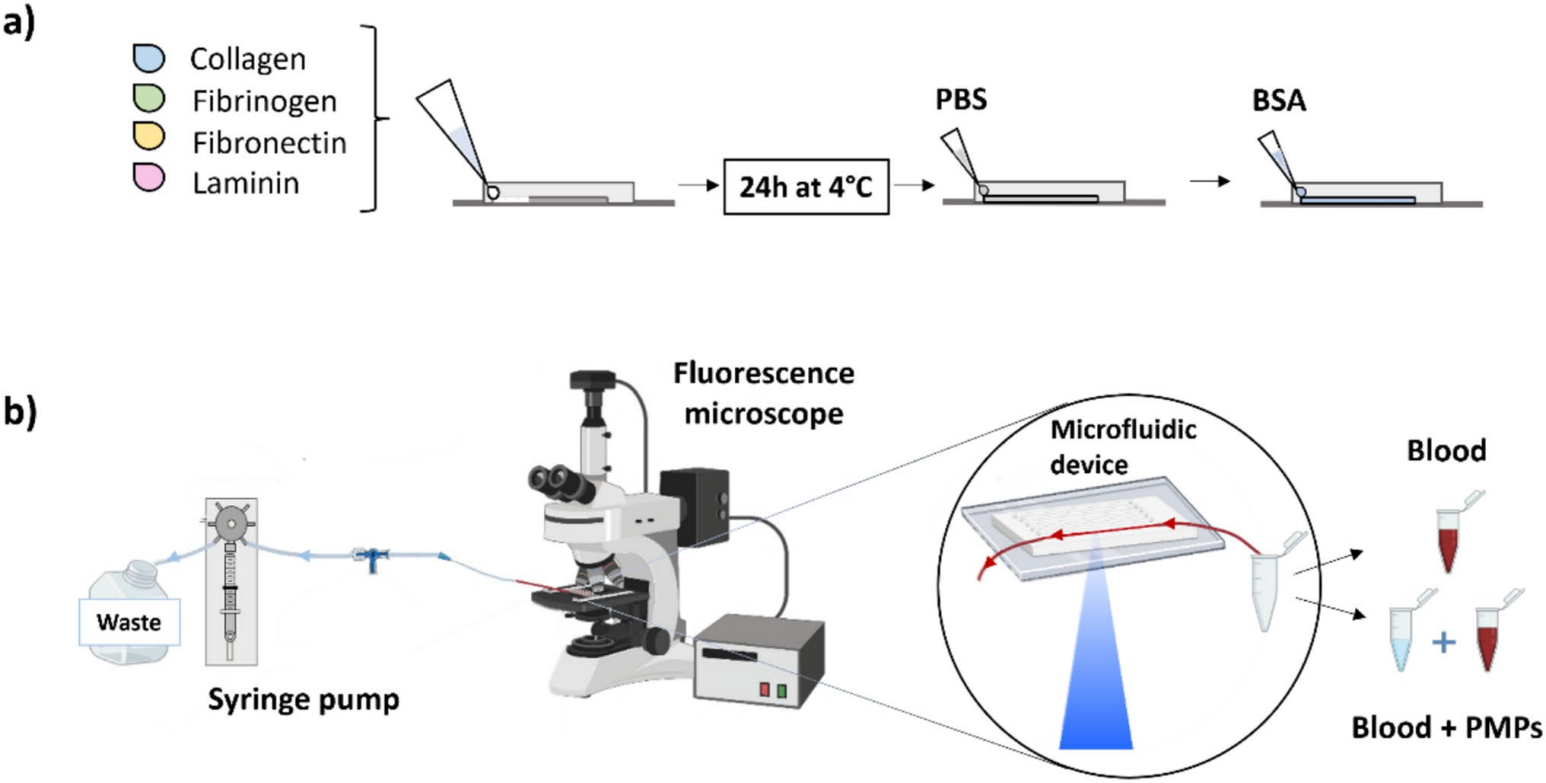
Experimental system, protocol and set up. **a** Microfluidic device and channel coating protocol with different extracellular matrix proteins; **b** experimental set up. PBS: phosphate buffer saline. BSA: bovine serum albumin. PDMPs: platelet-derived microparticles

The microfluidic platforms were fabricated by replica moulding of polydimethylsiloxane (PDMS) and subsequent sealing on 25 × 50 mm cover glasses via plasma bonding. Each platform featured 6 parallel microchannels (width = 1000 μm, height = 100 μm, length = 3 cm) [61]. Individual microchannels were loaded with ECM protein solutions as follows: 5 μL aliquots of 0.1 mg/mL collagen from equine tendon (Type I, CHRONO-LOG), human fibronectin (Sigma-Aldrich), human fibrinogen or mouse laminin (both from MilliporeSigma) were introduced through the microchannel outlet, covering one-third of the microchannel length, and incubated overnight at 4 °C (Fig. 1a). In a previous work, to validate our microfluidic system, we optimised the protein concentration to be used as substrate using collagen type I [61]. After incubation, the microchannels were rinsed with 50 μL of phosphate buffer saline (PBS, pH 7.4, Gibco™), and blocked with 1% BSA in PBS for 30 minutes at room temperature to prevent nonspecific bindings. Microfluidic devices with immobilized ECM proteins were stored at 4 °C and used the following day.

### Assembly and Operation of the Perfusion System, Image Acquisition and Analysis

To investigate platelet adhesion under flow, the microfluidic device was connected to a high-precision Hamilton PSD8 syringe pump (Hamilton) via non-adhesive Tygon^®^ tubing and placed under a Nikon Eclipse Ni fluorescence microscope equipped with a Nikon D-Qi2 digital camera (Figure 1b). Hirudin-anticoagulated, DiOC_6_-stained blood was perfused through ECM-coated microchannels at defined shear rates (300, 1100 or 1600 s^−1^) for 4 minutes at room temperature. Estimated wall shear stresses correspond to 13.5, 49.5 and 72 dyne/cm^2^, respectively.

For PDMP-enriched blood experiments, PDMPs obtained from one healthy donor’s blood were used; whole blood was premixed with the PDMP-rich fraction in a 10:1 v/v ratio and incubated for 10 minutes before being loaded into the perfusion system.

Platelet and PDMP adhesion was visualized using fluorescence microscopy (Zeiss, Axiovert,10x objective) and resulting images were analysed with a custom-made MATLAB script (MathWorks). Platelet aggregates were segmented using the Otsu thresholding algorithm; in < 5% of cases, where Otsu binarization was not successful, manual thresholding was applied. To distinguish single adherent platelets from aggregates, we calculated the expected area of one platelet (considering average diameter = 3 μm) based on the pixel size of the camera and the optical magnification. Regions corresponding to this area, and with fluorescence intensity above the identified threshold, were excluded from downstream analyses.

### Critical Endpoint Variables

Four parameters were evaluated to quantify platelet adhesion, aggregation and thrombus formation: 1. surface coverage (SC) representing the percentage of surface area covered by platelet thrombi; 2. number of thrombi (Nth) indicating the number of platelet adhesion sites on the ECM-covered surface; 3. mean thrombus area (Ath), i.e. the average size of the individual thrombi; 4. mean fluorescence intensity (FI) serving as a proxy for thrombus height.

### Statistical Analysis

Statistical analysis of the results was performed using GraphPad Prism 8 (GraphPad Software Inc.). Normal distribution of data was tested with the Shapiro–Wilk normality test. T-test or One-way Analysis of Variance (ANOVA) test was used when the normality hypothesis was satisfied. Conversely, Wilcoxon test or non-parametric Kruskal–Wallis test was performed. Statistical significance was assumed for p-values lower than 0.05.

## Results

### Platelet Adhesion, Aggregation and Thrombus Formation on Collagen, Fibrinogen, Fibronectin and Laminin Over Increasing Shear Stress

**Fig. 2:**
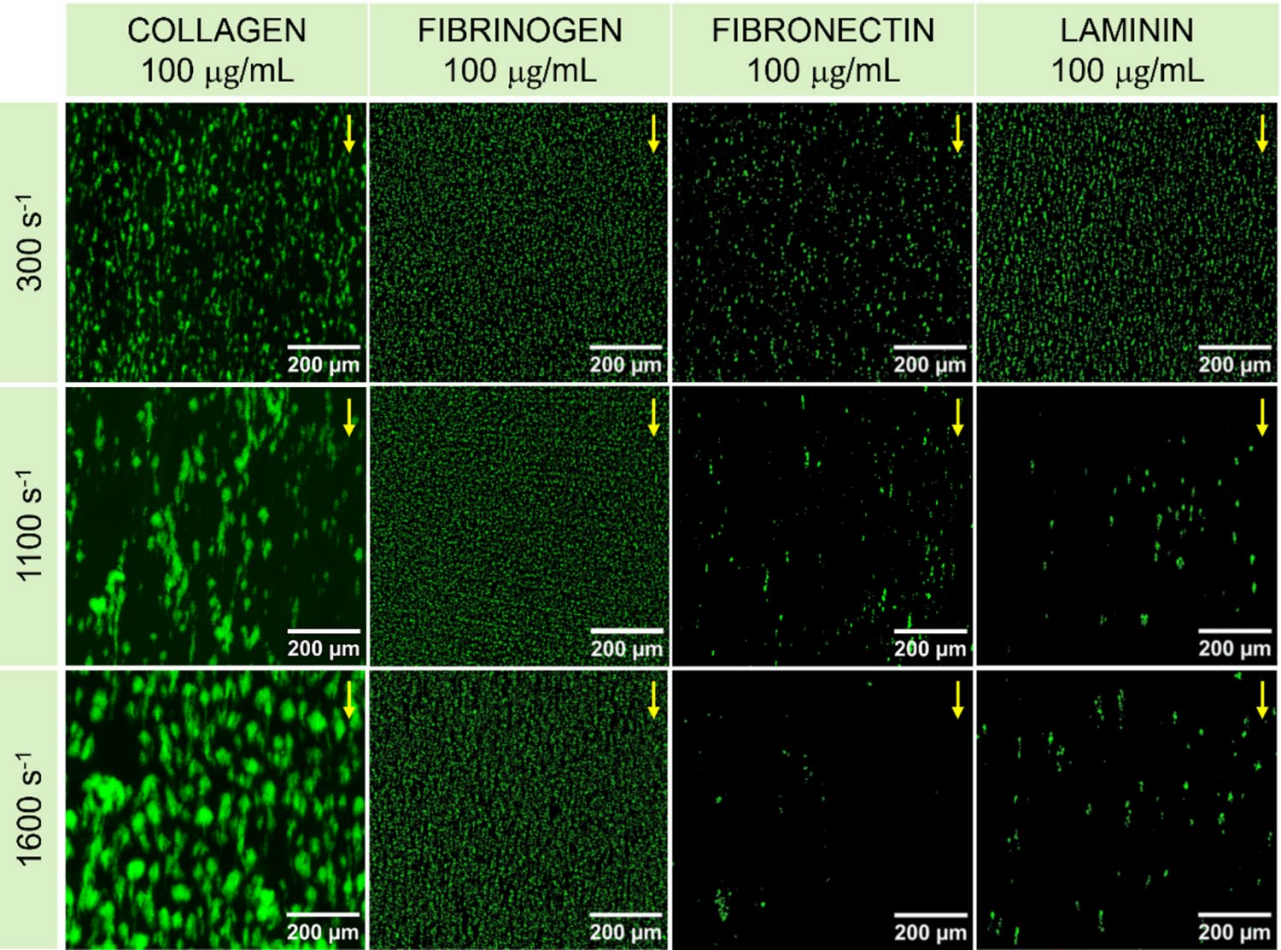
Representative images of platelet adhesion over tested protein substrates (collagen, fibrinogen, fibronectin, laminin) at 100 μg/mL concentration, at different shear rates (300, 1100, 1600 s^-1^), after 4 minutes of blood perfusion. Platelets were labelled with DiOC6 and imaged via fluorescence microscopy with 10x objective. Yellow arrows indicate the direction of flow

Using our methodology, platelet adhesion and aggregation could be detected for all four ECM proteins tested under a range of flow conditions. A representative image of platelet adhesion and aggregation under flow conditions for four ECM proteins–collagen, fibrinogen, fibronectin, and laminin–under venous, arterial and pathological blood flow conditions–300, 1100, and 1600 s^−1^– is shown in Fig. 2.

Of the ECM substrates tested, collagen exhibited the greatest degree of platelet adhesion at each applied shear rate. Compared to the other ECM substrates it was the only substrate that supported increased thrombus formation as shear rate magnitude increased. In contrast, platelet adhesion and aggregation on fibrinogen appear unaffected by shear rate magnitude up to 1600 s^−1^, while fibronectin and laminin exhibited a negative correlation between thrombus formation and shear rate.

Given the marked thrombogenic response observed on collagen, time-dependent platelet adhesion and aggregation kinetics on this substrate were additionally evaluated (Supplementary Figure S1), illustrating the temporal trends of the measured thrombus formation parameters at the three shear rates. For all shear rates, surface coverage did not reach saturation over this timescale. Fluorescence intensity exhibited an approximately linear increase throughout the experiment across all tested shear rate levels, likely indicating a progressive growth of thrombus height. At 1100 and 1600 s^−1^ the number of thrombi decreased substantially while mean thrombus area increased swiftly, suggesting the merging of adjacent thrombi.

### Platelet-Derived Microparticle Characterization

Microparticles generated by sonication ranged in size from 280 to 550 nm, with minimal variation, as indicated by a polydispersity index of 0.15. The particles were predominantly dense, opaque, and filled in appearance (>60% per 1200x field), although some exhibited lighter staining or appeared as intact membrane vesicles. Rare, fragmented vesicles were observed by transmission electron microscopy (3000x magnification).

**Fig. 3:**
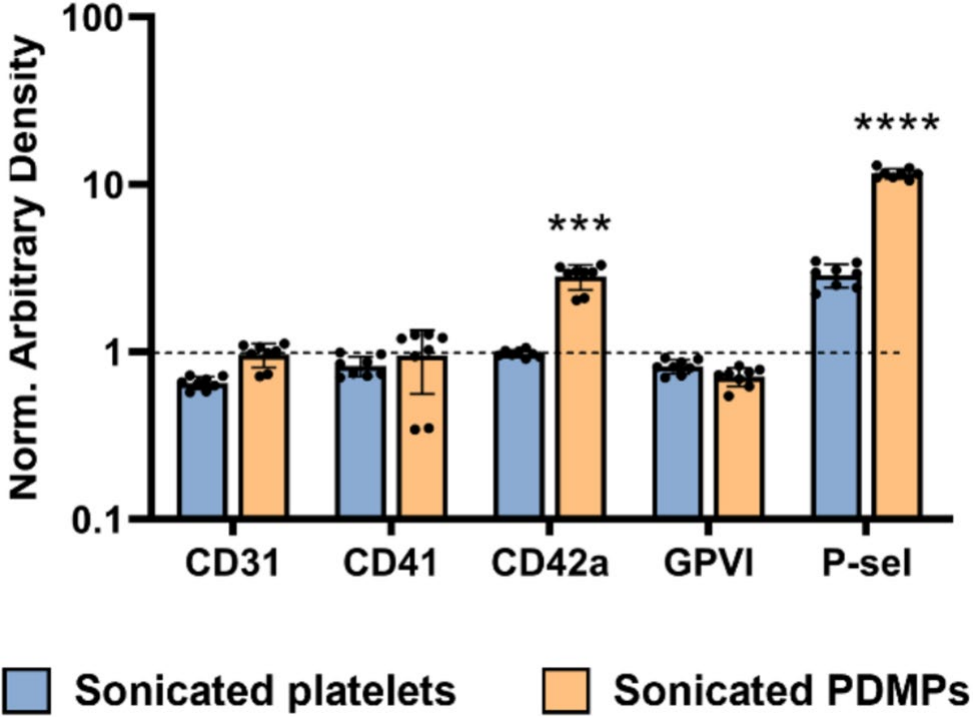
Surface expression of adhesion receptors on sonicated platelets (blue) and platelet-derived microparticles (orange) expressed as normalised arbitrary density (logarithmic scale), obtained as ratio of receptors density of interest over resting platelets’ receptors density. Investigated adhesion receptors: CD31 (PECAM), CD41 (GPIIb), CD42a (GP IX), GPVI and P-sel (P-selectin). n = 8. Data are reported as mean ± standard error of mean (SEM) and were analysed by Kruskal-Wallis test. Statistical significance is reported relative to resting platelets. ***p < 0.001, ****p < 0.0001

The density of adhesion receptors’ expression on sonicated platelets and sonicated PDMPs, normalised over receptors’ density of resting platelets, is shown in Fig. 3. Density of expression of CD31 (i.e. PECAM), CD41 (i.e. GP IIb), and GP VI, both on sonicated platelets and PDMPs, was comparable to that of resting platelets. In contrast, PDMPs showed a 2.8-fold increase in expression density of CD42a, i.e. GP IX, part of the GP Ib-IX-V complex (*p* < 0.001), and a 11.7-fold increase in the expression density of P-selectin (*p* < 0.0001). Sonicated platelets also showed a higher density of P-selectin expression versus resting platelets, but without statistical significance.

#### Collagen Substrate–Effect of Shear and Platelet-Derived Microparticles on Thrombus Formation

**Fig. 4:**
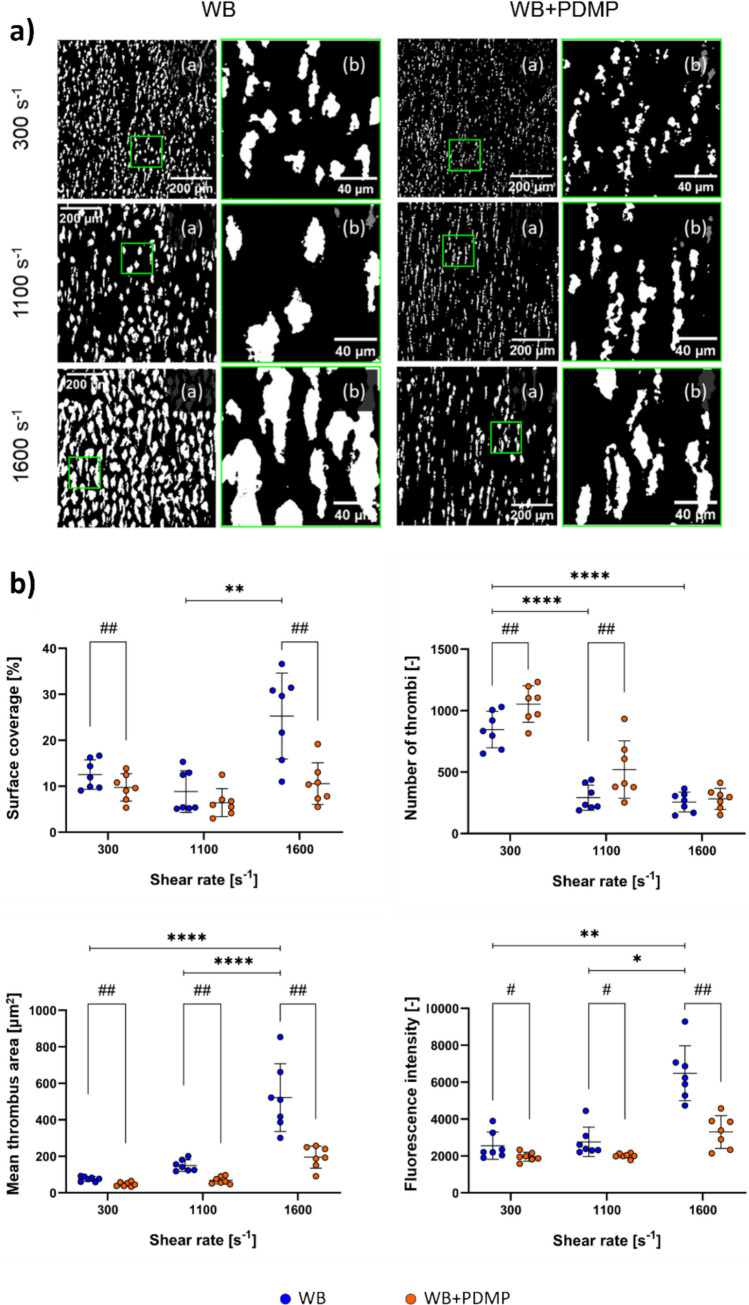
Thrombus formation on 100 μg/mL collagen substrate in control whole blood samples and PDMP-enriched whole blood samples. **a** Representative images of platelet adhesion on collagen substrate without (left) or with (right) PDMP addition to whole blood. **b** Thrombus formation for three shear rate levels (300, 1100, 1600 s^−1^) comparing whole blood samples (blue) vs. whole blood + PDMPs (orange), expressed in terms of surface coverage, number of thrombi, mean thrombus area and mean fluorescence intensity. n = 7. Data are reported as mean ± standard deviation. Differences across shear rates were analysed by ordinary one-way ANOVA or Kruskal-Wallis test, depending on the normality of data distribution. *p < 0.05, **p < 0.01, ****p < 0.0001. Between-group comparisons at each shear rate were performed using paired t-test or Wilcoxon test, depending on the normality of the distribution. #*p* < 0.05, ##*p* < 0.01

Thrombus formation conducted on collagen substrates, perfusing both WB and WB + PDMPs, is shown in Fig. 4.

##### Shear Effects

Whole blood exposure to increasing shear rates facilitated platelet adhesion to collagen, as reflected by increased SC, Ath and FI. These trends are qualitatively evident in the representative, binarized images (Fig. 4a, left). Quantification of data obtained by image analysis is reported in Fig. 4b (blue data points). Surface coverage increased from 12.6% ± 3.2% to 25.3% ± 9.3% at 300 and 1600 s^−1^, respectively. Correspondingly, the area of thrombi increased from 76.6 ± 13.2 mm^2^ to 522.0 ± 186.1 mm^2^ at 300 and 1600 s^−1^, respectively, and fluorescence intensity increased from 2555 ± 738 to 6482 ± 1486 at 300 and 1600 s^−1^. In contrast, the number of thrombi decreased from 846 ± 148 to 256 ± 81, at 300 and 1600 s^−1^, respectively, with increasing shear rate, suggesting that adjacent thrombi fuse together forming fewer but larger thrombi (Fig. 4b).

##### Influence of Platelet-Derived Microparticles on Platelet Adhesion, Aggregation and Thrombus Formation

Representative, binarized images showing the effect of PDMP addition to WB on platelet adhesion, aggregation and thrombus formation on collagen are reported in Fig. 4a on the right. Addition of circulating PDMPs had an inhibitory effect reducing platelet aggregation on collagen, with a decrease in surface coverage, area of thrombi and fluorescence intensity, but not number of thrombi, across all shear stress magnitudes. PDMP addition led to surface coverage of 9.8% ± 3.0% and 10.6% ± 4.5% at 300 and 1600 s^−1^, respectively, an overall decrease of coverage of 22.3 and 58.1%, respectively, compared to the no PDMP whole blood condition (*p* < 0.01) (Figure 4b). PDMP addition resulted in area of thrombi of 47.6 ± 11.9 mm^2^ and 195.9 ± 60.3 mm^2^ at 300 and 1600 s^−1^, respectively. This corresponded to an overall decrease of area of 37.9 and 62.5%, compared to the no PDMP whole blood condition at 300 and 1600 s^−1^ (*p* < 0.01). PDMP addition led to fluorescence intensity of 1961 ± 243 and 3301 ± 888 at 300 and 1600 s^−1^, respectively. This corresponded to an overall decrease of FI of 23.3% and 49.1%, compared to the no PDMP whole blood condition at 300 and 1600 s^−1^ (*p* < 0.05 and *p* < 0.01). The difference reached statistical significance at every shear rate magnitude, except for SC at 1100 s^−1^ and Nth at 1600 s^−1^. A nearly 2-fold decrease in SC, Ath and FI was observed when WB was spiked with PDMPs at 1600 s^−1^ (*p* < 0.01). Notably, adding PDMPs to whole blood significantly increased the number of thrombi, or sites of adhesion, only at lower shear magnitudes (*p* < 0.01 at 300 and 1100 s^−1^).

#### Fibrinogen Substrate–Effect of Shear and Platelet-Derived Microparticles on Thrombus Formation

**Fig. 5:**
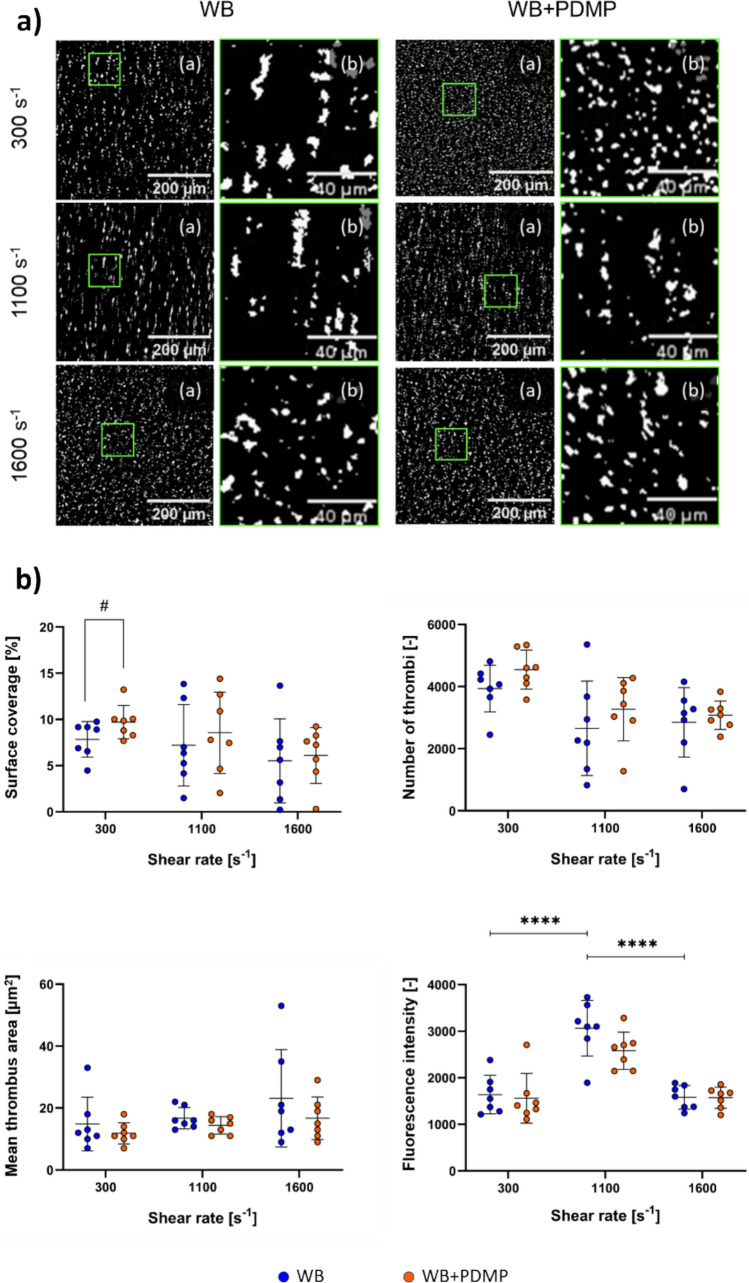
Thrombus formation on 100 μg/mL fibrinogen substrate in control whole blood samples and PDMP-enriched whole blood samples. a) Representative images of platelet adhesion on fibrinogen substrate without (left) or with (right) PDMP addition to whole blood. b) Thrombus formation for three shear rate levels (300, 1100, 1600 s^−1^) comparing whole blood samples (blue) vs. PDMP-enriched whole blood (orange), expressed in terms of surface coverage, number of thrombi, mean thrombus area and mean fluorescence intensity. n = 7. Data are reported as mean ± standard deviation. Differences across shear rates were analysed by ordinary one-way ANOVA or Kruskal-Wallis test, depending on the normality of data distribution. ****p < 0.0001. Between-group comparisons at each shear rate were performed using paired t-test or Wilcoxon test, depending on the normality of the distribution, for each shear rate. #*p* < 0.05

Thrombus formation conducted on fibrinogen substrates, perfusing both WB and WB + PDMPs, is shown in Fig. 5.

##### Shear Effects

Figure 5a, left, reports representative, binarized images of platelets adhered on fibrinogen substrate after perfusing WB for 4 minutes. Fibrinogen induced nearly constant thrombus formation throughout the range of tested shear magnitudes, although with a slightly decreasing trend, as evidenced by sustained levels of SC, Nth and Ath. An exception was observed in FI at 1100 s^−1^, where a peak at 3065 ± 597 led to a significant difference from the other two conditions (*p* < 0.0001), where FI reached 1637 ± 414 and 1580 ± 255 at 300 and 1600 s^−1^, respectively (Fig. 5b, blue data points).

##### Influence of Platelet-Derived Microparticles on Platelet Adhesion, Aggregation and Thrombus Formation

Figure 5a, right, shows representative, binarized images of platelet adhesion on fibrinogen in WB + PDMP samples.

PDMP addition to WB perfused on fibrinogen led to a different behaviour compared to collagen: here, the minor, non-significant increase in the number of thrombi in the WB + PDMPs samples was counterbalanced by only a slight reduction in thrombus area. In fact, PDMP addition resulted in area of thrombi of 12.0 ± 3.6 mm^2^ at 1100 s^−1^, corresponding to an overall decrease of 32.3% compared to the no PDMP whole blood condition (17.7 ± 7.6 mm^2^, *p* < 0.05), whereas no significant reduction was observed at the other shear rates (Fig. 5b). Therefore, surface coverage levels in presence of PDMP fraction were comparable to no PDMP whole blood results, except for a slight increase at 300 s^−1^, where SC reached 9.7% ± 1.8%, corresponding to an overall increase of 23.7% compared to the no PDMP whole blood condition (7.8% ± 1.9%, *p* < 0.05).

#### Fibronectin Substrate–Effect of Shear and Platelet-Derived Microparticles on Thrombus Formation

**Fig. 6:**
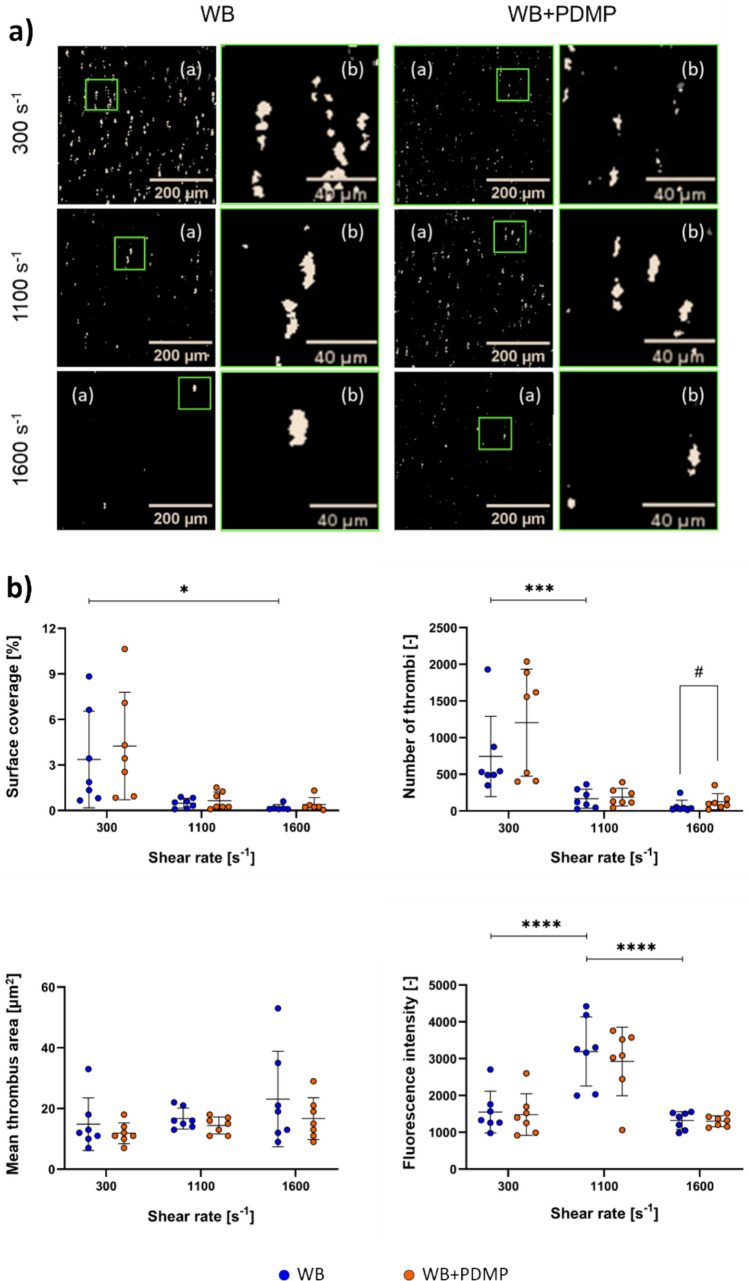
Thrombus formation on 100 μg/mL fibronectin substrate in control whole blood samples and PDMP-enriched whole blood samples. a) Representative images of platelet adhesion on fibronectin substrate without (left) or with (right) PDMP addition to whole blood. c) Thrombus formation for three shear rate levels (300, 1100, 1600 s^−1^) comparing whole blood samples (blue) vs. whole blood + PDMP (orange), expressed in terms of surface coverage, number of thrombi, mean thrombus area and mean fluorescence intensity. n = 7. Data are reported as mean ± standard deviation. Differences across shear rates were analysed by ordinary one-way ANOVA or Kruskal-Wallis test, depending on the normality of data distribution. *p < 0.05, ***p < 0.001, ****p < 0.0001. Between-group comparisons at each shear rate were performed using paired t-test or Wilcoxon test, depending on the normality of the distribution, for each shear rate. #*p* < 0.05

Thrombus formation conducted on fibronectin substrates, perfusing both WB and WB + PDMPs, is shown in Fig. 6.

##### Shear Effects

WB exposure to increasing shear rates inhibited platelet adhesion to fibronectin, with reduced SC and Nth, as displayed by representative images in Fig. 6a, left, and confirmed by image analysis-based quantification (Fig. 6b, blue data points). Surface coverage decreased from 3.4% ± 3.2% to 0.2% ± 0.2% at 300 and 1600 s^−1^, respectively (*p* < 0.05). Correspondingly, the number of thrombi decreased from 745 ± 547 to 63 ± 83 at 300 and 1600 s^−1^, respectively (*p* < 0.001). Similar to fibrinogen, FI showed a peak of 2926 ± 931 at 1100 s^−1^, causing a statistically significant difference compared to the other two shear conditions (*p* < 0.001 compared to both 300 and 1600 s^−1^).

##### Influence of Platelet-Derived Microparticles on Platelet Adhesion, Aggregation and Thrombus Formation

Figure 6a, right, shows representative, binarized images of platelet adhesion on fibronectin in WB + PDMP samples. Figure 6b (orange data points) reports quantification of data computed by image analysis.

Addition of PDMP did not exert significant effects on thrombus formation on fibronectin. A slightly increasing trend for platelet adhesion was observed, but only the number of thrombi at 1600 s^−1^ reached statistical significance (*p* < 0.05), going from 63 ± 83 to 126 ± 111 for WB and WB + PDMP, respectively.

#### Laminin Substrate–Effect of Shear and Platelet-Derived Microparticles on Thrombus Formation

##### Shear Effects

**Fig. 7:**
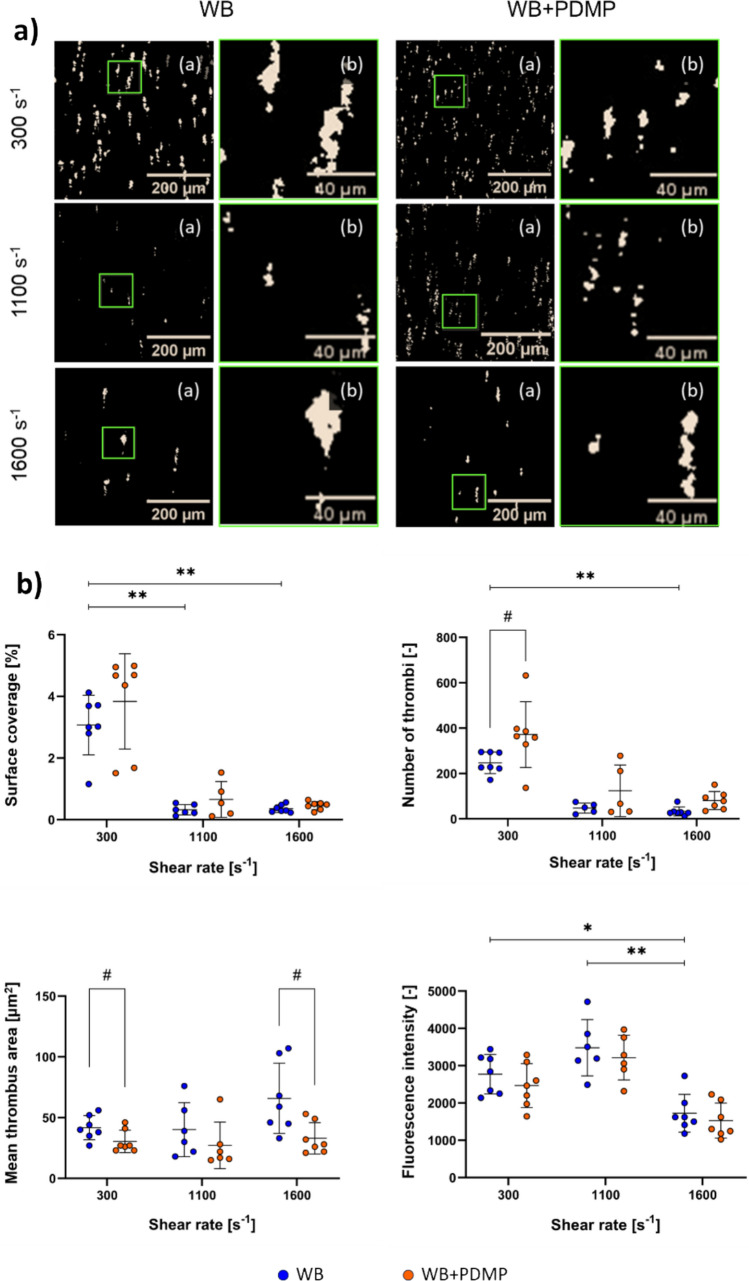
Thrombus formation on 100 μg/mL laminin substrate in control whole blood samples and PDMP-enriched whole blood samples. **a** Representative images of platelet adhesion on laminin substrate without (left) or with (right) PDMP addition to whole blood. **b** Thrombus formation for three shear rate levels (300, 1100, 1600 s^−1^) comparing whole blood samples (blue) vs. whole blood + PDMP (orange), expressed in terms of surface coverage, number of thrombi, mean thrombus area and mean fluorescence intensity. n = 7. Data are reported as mean ± standard deviation. Differences across shear rates were analysed by ordinary one-way ANOVA or Kruskal-Wallis test, depending on the normality of data distribution. *p < 0.05, ***p < 0.001. Between-group comparisons at each shear rate were performed using paired t-test or Wilcoxon test, depending on the normality of the distribution, for each shear rate. #*p* < 0.05

On laminin, which is a link protein of the ECM, thrombus formation showed a trend similar to fibronectin for all the parameters (Fig. 7).

WB exposure to increasing shear rates resulted in inhibition of platelet adhesion to laminin. Surface coverage decreased from 3.07% ± 0.97% at 300 s^−1^ to 0.36% ± 0.15% at 1100 s^−1^ (*p* < 0.01), with a further decline to 0.36% ± 0.12% at 1600 s^−1^ (*p* < 0.01 compared to 300 s^−1^) (Fig. 7b, blue data points).

The significant reduction of surface coverage for increasing shear rate was mainly associated to a decrease in the number of thrombi. In fact, Nth decreased from 247 ± 48 at 300 s^−1^ to 33 ± 20 at 1600 s^−1^ (*p* < 0.01). No significant differences in thrombus size could be observed, while fluorescence intensity, indirectly representing thrombus height, showed a decrease at 1600 s⁻^1^, falling to 1528 ± 469, leading to statistically significant differences with FI at 300 s^−1^ (2467 ± 590, *p* < 0.05) and 1100 s^−1^ (3217 ± 598, *p* < 0.01).

##### Influence of Platelet-Derived Microparticles on Platelet Adhesion, Aggregation and Thrombus Formation

Thrombus formation on laminin in WB, in presence of PDMPs, is shown qualitatively in Fig. 7a, right. Quantification reveals that PDMP addition exerted effects on platelet adhesion and aggregation on laminin similar to those observed on fibronectin, with a slightly increasing trend for surface coverage, though without reaching statistical differences (Fig. 7b). PDMP-enriched WB samples showed an increase in number of thrombi, reaching statistical significance (*p* < 0.05) at 300 s^−1^, going from 247 ± 48 to 371 ± 145 for WB and WB + PDMP samples, respectively, corresponding to an overall increase of 50.4%. Concurrently, PDMP addition led to area of thrombi of 30.4 ± 9.2 mm^2^ and 33.0 ± 12.9 mm^2^ at 300 and 1600 s^−1^, respectively, equivalent to an overall decrease of area of 27.1 and 49.9%, respectively, compared to the no PDMP whole blood condition (*p* < 0.05).

FI did not show any significant change following PDMP addition, suggesting that thrombus height was not affected.

Table 2 summarizes the effect exerted by PDMP addition to whole blood on thrombus formation on the four tested substrates, expressed as percentage of variation of the PDMP-enriched samples compared to the control samples, for each parameter extracted by image analysis.

**Table 2:** Effect of PDMP addition to whole blood on thrombus formation. Percentage of variation of the four parameters computed by image analysis and corresponding p-values (when significant) compared to control group

		300 s^−1^	1100 s^−1^	1600 s^−1^
Collagen	SC	− 22.3% *p < 0.01*	− 27.3%	− 58.1% *p < 0.01*
	Nth	24.6% *p < 0.01*	78.3% *p < 0.01*	10.4%
	Ath	− 37.9% *p < 0.01*	− 54.3% *p < 0.01*	− 62.5% *p < 0.01*
	FI	− 23.3% *p < 0.05*	− 27.1% *p < 0.05*	− 49.1% *p < 0.01*
Fibrinogen	SC	23.7% *p < 0.05*	18.9%	10.6%
	Nth	19.8%	23.1%	8.0%
	Ath	− 15.3%	− 32.3% *p < 0.05*	7.5%
	FI	− 4.67%	− 15.7%	− 0.6%
Fibronectin	SC	26.4%	33.6%	106.1%
	Nth	61.8%	13.3%	98.9% *p < 0.05*
	Ath	− 20.2%	− 13.7%	− 27.8%
	FI	− 4.5%	− 8.4%	− 1.6%
Laminin	SC	24.9%	81.8%	27.2%
	Nth	50.43% *p < 0.05*	159.4%	146.5%
	Ath	− 27.05% *p < 0.05*	− 32.4%	− 49.89% *p < 0.05*
	FI	− 11.03%	− 7.6%	− 11.4%

SC: surface coverage. Nth: number of thrombi. Ath: mean area of thrombi. FI: mean fluorescence intensity. Absence of p value implies data were not statistically significant at the p < 0.05 level.

## Discussion

In vascular damage a dynamic interplay exists between platelets and circulating platelet-derived microparticles and the underlying exposed vessel wall ECM proteins. To date, these dynamic interactions have been incompletely defined. Here, using four extracellular matrix protein substrates, we systematically examined platelet adhesion, aggregation and thrombus formation, under varying flow/shear conditions. Further, for each of the conditions studied we examined the effect of addition of platelet-derived microparticles as modulators of the thrombotic processes. Overall, our findings reveal a clear hierarchy of platelet adhesivity and thrombus formation among ECM substrates, with collagen > fibrinogen > fibronectin > laminin. Each protein supported distinct qualitative and quantitative patterns of platelet adhesion, aggregation, and thrombus formation, potentially driven by differences in receptor-binding motifs, platelet-activating capacity, and bonding stability under shear forces. Increasing shear rate consistently amplified pro-thrombotic behaviour, particularly on collagen, where GP VI- and α2β1-mediated activation enabled robust platelet recruitment under high flow. Finally, across all substrates and shear conditions, the addition of platelet-derived microparticles altered thrombus formation, affecting both thrombus morphology and structure.

### Effect of Underlying Substrate in the Context of Varying Shear

For all the ECM proteins tested, immobilised collagen supported the highest levels of platelet adhesion and aggregation, exhibiting an increase with increasing shear rates. Our results demonstrating increasing platelet accumulation on collagen, as to surface coverage, thrombus area and thrombus height (FI) for increasing shear rate, are consistent with those reported by others [6, 61–63], despite other groups reporting different shear values for peak thrombotic effects.

Firm platelet adhesion under flow can only occur when the bond strength between the platelet and substrate exceeds the rheological forces that oppose and stress their interaction. Substantial thrombus formation on collagen at high shear rates is possible because, in addition to VWF–GP Ibα binding, collagen also binds GP VI on platelets, facilitated by α2β1. This enables platelet rolling on the vessel surface under high shear stress conditions and promotes platelet activation, thereby enhancing platelet recruitment and aggregation via αIIbß3 [64].

Unlike collagen, fibrinogen primarily acts as a bridging molecule between activated platelets. Although platelet accumulation at 300 and 1100 s^−1^ on collagen and fibrinogen is comparable in terms of SC and FI, our results reveal a higher number of thrombi with notably smaller surface areas on fibrinogen. This finding is in accordance with Zaidi et al. [65], who observed a prevalence of small thrombi in platelet deposition on fibrinogen when compared to collagen at 800 s^−1^, along with poor platelet aggregation. In addition, we found a decreasing trend for SC with increasing shear rate on fibrinogen, although non-statistically significant, as supported by Savage et al., who observed an impediment to sustaining platelet accumulation on fibrinogen at high shear rates [66]. Platelet-fibrinogen binding is mainly mediated by αIIbß3 integrin and only in second instance by GP VI: several studies underlie the necessity of this integrin to undergo a conformational change and become activated (high-affinity state) for a stable bond, thus physiological agonists such as adenosine diphosphate (ADP) or thrombin are required, conversely to collagen, which is able to activate platelets itself [67, 68]. Without an integrin activator, platelets extend filipodia and only scarcely spread lamellipodia when flowing over a fibrinogen substrate, causing platelet mobility attenuation, but not block [69]. This behaviour likely explains our findings of non-sustained platelet adhesion on fibrinogen for increasing shear rate.

Fibronectin, a key ECM protein, has been shown to contribute to platelet adhesion at the early stages of coagulation. While it plays an important role in stabilizing thrombi, previous studies indicate that it supports only moderate platelet adhesion [70], forming less organized thrombi compared to other matrix proteins, such as collagen [71]. Under static conditions, fibronectin supports platelet adhesion and spreading for isolated platelets via α5ß1, αvß3 and αIIbß3, with only the latter integrin able to activate platelets. αIIbß3-dependent adhesion in plasma requires prior stimulation, demonstrating that plasma provides an anti-adhesive medium, which prevents adhesion in the absence of stimulating agents [72]. In our experiments, the mechanical stimulation exerted by flow induced platelets to partially adhere to fibronectin only at the lowest level of shear tested. At shear rates of 1100 and 1600 s^−1^, platelet accumulation nearly ceased, with only small and sparse platelet aggregates observed, suggesting that no mechanisms were effective in facilitating platelet rolling and adhesion to the immobilized protein. These results are in agreement with Maurer et al. [73], who found an increase in platelet adhesion and thrombus volume only up to 500 s^−1^, followed by a reduction for higher shear rates. A possible explanation for this can be found in Bastida et al. [74], who suggested that fibronectin alone cannot provide a platelet-protein binding strong enough to resist high shear forces via its preferred integrin α5ß1. Under these conditions, it requires the contribution of other adhesive proteins such as VWF, to support platelet adhesion, through fibronectin–GP Ib binding. In addition, it has been proposed that immobilised fibronectin may be inefficient because it is not assembled in supramolecular structures [75].

Laminin provided the lowest level of platelet accumulation among the tested substrates. Like fibronectin, SC dropped nearly to zero at the medium and high shear rates, with Nth, Ath and FI also showing a comparable trend. However, thrombus formation on laminin spanned a different range in terms of number and mean area of the thrombi: Nth was reduced and Ath increased when compared to fibronectin, particularly at 300 s^−1^. Our findings are partially in accordance with Hindriks et al. [76], who showed maximal platelet adhesion on laminin between 300 and 800 s^−1^, followed by a reduction of SC for increasing shear rate. In contrast, Inoue et al. [77] showed a percent surface coverage on laminin comparable to the one on collagen at 1500 s^−1^ shear rate.

It has been established that this protein interacts with GP VI, and that this interaction is facilitated by integrin α6ß1 with a mechanism similar to the one of collagen and α2ß1 [78]. Nonetheless, the affinity between GP VI and laminin is much lower than that of collagen [76] and this might explain our reduced results for laminin. Indeed, despite the high SC, even Inoue et al. observed relatively small aggregates at 1500 s^−1^ on laminin surface, indicating that platelets were not strongly activated. This observation is in agreement with early works of Ill et al., who observed lack of serotonin release and an intact, round appearance of laminin-bound platelets [79]. As the shear rate (and thus the flow rate) increases, the time window during which platelet receptors and immobilised proteins come into contact shortens. Only those bindings known to be efficient under high flow rate remain effective, helping platelet rolling and tethering, such as ECM-bound VWF-GP Ib-IX-V [80].

It has been suggested that at the higher shear rates typically found in the arteriolar circulation, both fibronectin [70] and laminin in the ECM [77] interact with VWF in a manner similar to collagen, indirectly supporting GP Ibα-mediated platelet tethering. However, our results do not support this hypothesis. On the contrary, we found few adhesion sites at high shear stresses on laminin and fibronectin, although once the initial adhesion started, platelets were still able to aggregate grasping those adherent ones, in fact both thrombus size and height did not decrease. Other protein isoforms typically found in human vessel wall (e.g. laminin 411, 511, 521) should be investigated in the future to deepen this matter.

### Influence of Platelet-Derived Microparticles on Platelet Adhesion, Aggregation and Thrombus Formation

The addition of PDMPs to blood showed a clear effect on collagen, inducing a decrease in surface coverage, thrombus area and thrombus height (FI), though with a slight increase in the number of thrombi, which tend to be smaller, at low but not high shear. The increment in platelet adhesion may be due to the increased number of particles with a procoagulant surface in the blood samples spiked with PDMPs. We speculate that PDMPs, enriched with platelet surface receptors, may competitively occupy intact platelet binding sites, leading to a smaller thrombus area. Prior work has demonstrated that platelet and PDMP phenotypes differ substantially depending on whether activation is driven biochemically or mechanically [19, 59, 81]. Under shear stress, platelets undergo membrane scrambling with phosphatidylserine (PS) exposure, redistribution of membrane receptors, limited α-granule secretion, and minimal αIIbβ3 activation, a phenotype distinct from that induced by agonists such as ADP or thrombin. In the 2023 study, Roka-Moiia et al. [19] further showed that PDMPs generated through sonication, a model of extreme mechanical insult, exhibit strong phosphatidylserine enrichment, exposure of adhesive and procoagulant motifs and altered surface receptor composition consistent with mechanical membrane disruption. Together, these observations support the hypothesis that mechanically derived PDMPs may function as competitive antagonists for platelet binding to the ECM, by presenting receptor-enriched or pro-adhesive surfaces capable of occupying binding sites or altering local adhesive microenvironments.

In our experiments we employed a high concentration of hirudin to directly inhibit thrombin activity, effectively interrupting the coagulation feedback loop. By blocking thrombin generated on activated platelets and PDMPs, hirudin prevents fibrin formation as well as thrombin-mediated platelet activation, allowing us to isolate the platelet-microparticle interactions from secondary coagulation effects. In fact, although PS-exposing microparticles typically amplify thrombin generation, our hirudin-anticoagulated system isolates thrombin-independent functions of PS⁺ PDMPs. PS remains biologically active as a charged, pro-adhesive surface that promotes PDMP clustering and adsorption to the ECM protein substrates. Thus, the enhanced number of adhesion sites (Nth) we observed reflects thrombin-independent procoagulant and adhesive properties intrinsic to PS⁺ PDMPs, consistent with the membrane-scrambling phenotype described under mechanical activation [19]. Our hypothesis, nevertheless, requires further experimental verification. Future experiments employing Ca^2+^ chelation could help discriminate between initial platelet adhesion and subsequent calcium-dependent processes including platelet activation, integrin-mediated aggregation, thrombus growth and stabilization.

Our results disagree with Suades et al. [64], who observed an increase, even though non-significant, in platelet surface coverage on a collagen substrate at 1500 s^−1^ when comparing WB with WB + PDMPs; nonetheless, they found no difference in aggregation in suspension triggered by collagen. Consistent with our work, Roka-Moiia et al. [19] reported a decrease in platelet aggregation in suspension when WB spiked with PDMPs (obtained via sonication) was exposed to collagen, ADP or TRAP-6. Notably, in prior work we have observed that pre-exposure of damaged arterial wall to platelet-derived microparticles can limit subsequent platelet adhesion to the injury site in a dose-dependent fashion (Slepian et al. unpublished results). Consistently, our present results suggest that addition of PDMPs to WB prevents both platelets anchorage and aggregation to the ECM protein substrates tested. We point out that others too have noted increased PDMP adhesion to damaged endothelial cells and exposed ECM as well, consistent with our results [82].

Our results show that collagen provided the highest level of platelet adhesion and aggregation, as expected, and thrombus formation on this substrate was the most negatively affected by the presence of PDMPs. While thrombus area and height were affected across all the tested shear rates, the number of thrombi was significantly different only at the two lower shear rates. PDMPs might be less efficient in rolling and tethering over the protein surfaces than intact platelets and consequently be dragged away by the high rheological forces exerted under high flow rate conditions.

When adding PDMPs to WB, a similar but less evident effect was found on fibrinogen, fibronectin and laminin compared to collagen. A trend toward an increase in the number of thrombi is noted, though thrombi are generally smaller with reduced surface area and height. Merten et al. [82] suggested that platelet activation is a prerequisite for platelet binding to microparticles, likely because the binding is GP IIbIIIa-mediated, as also supported by previous findings [83, 84]. As is well-known, collagen is a potent platelet activator, whereas fibrinogen, fibronectin and laminin do not directly activate platelets, rather they act as support for thrombus formation. This could explain why collagen was the most affected, as platelet-PDMPs binding is favoured by this protein, leading to a more pronounced reduction in thrombus size and height.

In agreement with our findings, Artemenko et al. [85] reported that PDMPs (originated from activated platelets) were only weakly incorporated into forming thrombi on fibrinogen, concluding that they are likely to not exert any significant effect on thrombus formation in healthy individuals.

### Next Steps and Limitations

Flow-based platelet assays rely on a number of key experimental parameters, including shear conditions, substrate composition, blood handling, and analytical readouts, all of which must be carefully defined to ensure reproducibility and physiological relevance. In this context, standardization efforts by the Scientific and Standardization Committee (SSC) of the International Society on Thrombosis and Haemostasis have emphasized the importance of controlling these parameters to improve comparability across studies [12]. Among others, it has been recommended that adhesive substrates incorporate a combination of collagen and tissue factor to better mimic the subendothelial matrix [86]. In addition, a comprehensive assessment of thrombus formation under flow should ideally include not only platelet adhesion and aggregation, but also fibrin deposition, which is itself influenced by shear conditions. In the present study, we focused on platelet adhesion and aggregation on individual protein substrates in order to isolate their specific contributions. Future work could extend this approach to incorporate SSC recommendations.

Future studies are planned to investigate thrombus formation dynamically over time on all the selected proteins, to better characterize the sequence of events leading to the endpoint results, including potential thrombus detachment or contraction, or other morphological changes that may influence the final outcomes.

Our findings were obtained under a defined platelet-to-PDMP ratio and defined protein substrates concentration and may not directly extend to other conditions. Additional studies varying the relative proportions of platelets and PDMPs and the concentration of the proteins are needed to assess the robustness and generalizability of the observations. Furthermore, the specific roles of individual platelet adhesion receptors and the extent of receptor–ligand competition between platelets and PDMPs remain to be established. Targeted inhibition, receptor-blocking experiments or double labelling of platelets and PDMPs could provide valuable insights into the molecular mechanisms governing platelet adhesion and PDMP-mediated modulation of thrombus formation.

### Potential Mechanistic Implications of Our Findings

The distinct adhesion patterns observed across collagen, fibrinogen, fibronectin, and laminin suggest that platelet recruitment under flow is governed not only by receptor activation but potentially by the abundance of accessible integrin-recognition motifs within each substrate. The five principal platelet integrins, αIIbβ3 (GP IIbIIIa), α2β1, α5β1, α6β1, and αvβ3, bind a limited set of peptide motifs, most prominently the RGD sequence (recognized by αIIbβ3 and αvβ3), the GFOGER and related triple-helical motifs (α2β1/GP VI binding on collagen), PHSRN-RGD synergy sites (α5β1 on fibronectin), and SIEVL/laminin-α chain motifs (α6β1). Among the proteins studied, collagen contains no RGD but is densely enriched in GPO/GFOGER-like sequences, providing a very high stoichiometric density of platelet-activating sites. Fibronectin and fibrinogen contain multiple RGD motifs, while laminin carries few or none in accessible conformations, offering far lower effective ligand densities under flow. This framework may contribute to and explain selective adhesion: collagen’s high GP VI/α2β1 motif abundance yields dominant recruitment; fibrinogen and fibronectin provide intermediate αIIbβ3-mediated adhesion; laminin provides the fewest effective sites. PDMPs likely modulate this hierarchy through dual mechanisms, acting as decoys that compete for platelet integrin engagement while simultaneously serving as prothrombotic catalysts via surface phosphatidylserine, thus reshaping effective ligand stoichiometry and diminishing thrombus growth on high-affinity substrates such as collagen.

It is worthy to note that PDMP protein phenotype and contents strongly depend on the mechanism of microparticle formation [19, 20, 22]. PDMPs obtained *in vitro* by sonication appear to result from random fragmentation rather than shedding due to activation. These PDMPs are characterized by a high density of phosphatidylserine [19] and P-selectin and they express, among others, GP IIbIIIa, GP VI, GP Ibα and GP IX. We hypothesise that the higher density of some receptors (i.e. P-selectin and GPVI) contributes to alter platelet adhesion to ECM proteins observed in our work. PDMPs highly negatively charged surface can enhance thrombin generation by providing a catalytic surface for prothrombinase complex formation, thus increasing the prothrombin activation rate [87], but this was prevented by hirudin anticoagulation in our experiments. Nonetheless, PDMPs *in vivo* are also found in the blood of healthy individuals, suggesting they may play a role as sentinels or even have an anti-thrombotic function [88, 89]. Evidence exists regarding their ability to regulate coagulation through anticoagulant or fibrinolytic mechanisms. PDMPs have been found to carry TF pathway inhibitor on their surface and to promote the activity of the anticoagulant protein C [90, 91].

Accordingly, discordant results may be due to the lack of standardisation in PDMP isolation procedure, analysis and *in vitro* production, as well as to the existence of different subtypes of PDMPs, with heterogeneous size and content, and distinct roles [89, 92].

## Conclusions

Our findings further dissect and define the complex interplay of platelet adhesion and aggregation on specific extracellular matrix (ECM) proteins under flow conditions and highlight the modulatory role of platelet-derived microparticles in thrombus formation.

Collagen, the most potent substrate tested, supports the highest level of platelet adhesion and aggregation across all shear rates, with thrombus area, size and stability increasing as shear rate rises. In contrast, fibrinogen maintains a nearly constant thrombus formation, while both fibronectin and laminin exhibit declining adhesion and aggregation as shear rate rises, driven by a reduction in adhesion sites rather than thrombus size.

The addition of PDMPs to whole blood influences thrombus formation to a different extent depending on the substrate. On collagen, PDMPs inhibit thrombus formation, reflected in reduced thrombus area and fluorescence intensity, despite an increase in the number of adhesion sites at lower shear rates. Conversely, on fibrinogen, fibronectin, and laminin, PDMPs impact is less evident: they slightly enhance platelet adhesion by increasing the number of adhesion sites, meanwhile decreasing the mean thrombus area.

Our findings underscore the dynamic interplay between shear conditions, ECM proteins, and PDMPs in regulating thrombus formation. The distinct responses observed highlight the complexity of platelet adhesion and aggregation processes and provide a framework for future investigations into the molecular mechanisms governing PDMP-mediated modulation of haemostasis and thrombosis.

## Supplementary Information

Below is the link to the electronic supplementary material.

Supplementary file1 (DOCX 204 kb)

## Data Availability

The data that support the findings of this study are available from the corresponding authors upon reasonable request.

## References

[1] Roger, V. L., et al. Heart disease and stroke statistics-2011 update: a report from the American heart association. *Circulation.* 2011. 10.1161/CIR.0b013e3182009701. 10.1161/CIR.0b013e3182009701 PMC4418670 21160056

[2] Mozaffarian, D., et al. Heart disease and stroke statistics-2016 update: a report from the American heart association. *Circulation*. 133:38–360, 2015. 10.1161/CIR.0000000000000350. 10.1161/CIR.0000000000000350 26673558

[3] Arnold, J., A. Koyfman, and B. Long. High risk and low prevalence diseases: acute limb ischemia. *Am J Emergency Med*. 74:152–158, 2023. 10.1016/j.ajem.2023.09.052. 10.1016/j.ajem.2023.09.052 37844359

[4] Rivera, J., M. L. Lozano, L. Navarro-Núñez, and V. Vicente García. Platelet receptors and signaling in the dynamics of thrombus formation. *Haematologica.* 94(5):700–711, 2009. 10.3324/haematol.2008.003178. 19286885 10.3324/haematol.2008.003178 PMC2675683

[5] Jackson, S. P., W. S. Nesbitt, and S. Kulkarni. Signaling events underlying thrombus formation. *J Thromb Haemost*. 1(7):1602–1612, 2003. 10.1046/j.1538-7836.2003.00267.x. 12871297 10.1046/j.1538-7836.2003.00267.x

[6] Savage, B., F. Almus-Jacobs, and Z. M. Ruggeri. Specific synergy of multiple substrate-receptor interactions in platelet thrombus formation under flow. *Cell*. 94(5):657–666, 1998. 10.1016/S0092-8674(00)81607-4. 9741630 10.1016/s0092-8674(00)81607-4

[7] Janus-Bell, E., and P. H. Mangin. The relative importance of platelet integrins in hemostasis, thrombosis and beyond. *Ferrata Storti Foundation*. 2023. 10.3324/haematol.2022.282136. 10.3324/haematol.2022.282136 PMC10316258 36700400

[8] Watson, S. P., J. M. Auger, O. J. T. Mccarty, and A. C. Pearce. GPVI and integrin aIIbb3 signaling in platelets. *J Thromb Haemost*. 3:1752–1762, 2005. 16102042 10.1111/j.1538-7836.2005.01429.x

[9] Woulfe, D., et al. Signaling receptors on platelets and megakaryocytes. *Methods Mol. Biol.* 273:3–32, 2004. 10.1385/1-59259-783-1:003/COVER. 15308791 10.1385/1-59259-783-1:003

[10] Lee, D., K. P. Fong, M. R. King, L. F. Brass, and D. A. Hammer. Differential dynamics of platelet contact and spreading. *Biophys. J.* 102(3):472–482, 2012. 10.1016/j.bpj.2011.10.056. 22325269 10.1016/j.bpj.2011.10.056 PMC3274813

[11] Schneider, M. F., et al. Platelet adhesion and aggregate formation controlled by immobilised and soluble VWF. *BMC Mol Cell Biol*. 2020. 10.1186/s12860-020-00309-7. 10.1186/s12860-020-00309-7 PMC7488753 32917131

[12] Mangin, P. H., et al. In vitro flow based systems to study platelet function and thrombus formation: recommendations for standardization: communication from the SSC on Biorheology of the ISTH. *J Thromb Haemost*. 18(3):748–752, 2020. 10.1111/jth.14717. 32112535 10.1111/jth.14717

[13] Da, Q., M. Teruya, P. Guchhait, J. Teruya, J. S. Olson, and M. A. Cruz. Free hemoglobin increases von Willebrand factor-mediated platelet adhesion in vitro: implications for circulatory devices. *Blood*. 126(20):2338–2341, 2015. 10.1182/blood-2015-05. 26307534 10.1182/blood-2015-05-648030 PMC4643006

[14] Qi, Q. M., et al. In vitro measurement and modeling of platelet adhesion on VWF-coated surfaces in channel flow. *Biophys. J.* 116(6):1136–1151, 2019. 10.1016/j.bpj.2019.01.040. 30824114 10.1016/j.bpj.2019.01.040 PMC6428971

[15] Y.-P. Wu, P. G. De Groot, and J. J. Sixma. Shear stress-induced detachment of blood platelets from various surfaces. *American Heart Association*. 3202–3207, 1997. 10.1161/01.atv.17.11.3202 9409312

[16] Ruggeri, Z. M. Platelet adhesion under flow. *Microcirculation*. 16(1):58, 2009. 10.1080/10739680802651477. 19191170 10.1080/10739680802651477 PMC3057446

[17] Badimon, L., R. Suades, E. Fuentes, I. Palomo, and T. Padró. Role of platelet-derived microvesicles as crosstalk mediators in atherothrombosis and future pharmacology targets: A link between inflammation, atherosclerosis, and thrombosis. *Frontiers Media S.A*. 2016. 10.3389/fphar.2016.00293. 10.3389/fphar.2016.00293 PMC5005978 27630570

[18] Horstman, L. L., and Y. S. Ahn. Platelet microparticles: a wide-angle perspective. *Crit. Rev. Oncol. Hematol.* 30:111–142, 1999. 10439058 10.1016/s1040-8428(98)00044-4

[19] Roka-Moiia, Y., et al. Shear-mediated platelet microparticles demonstrate phenotypic heterogeneity as to morphology, receptor distribution, and hemostatic function. *Int. J. Mol. Sci.* 2023. 10.3390/ijms24087386. 10.3390/ijms24087386 PMC10138836 37108551

[20] El-Gamal, H., A. S. Parray, F. A. Mir, A. Shuaib, and A. Agouni. Circulating microparticles as biomarkers of stroke: A focus on the value of endothelial- and platelet-derived microparticles. *J. Cell. Physiol.* 234(10):16739–16754, 2019. 10.1002/jcp.28499. 30912147 10.1002/jcp.28499

[21] Perez-Pujol, S., P. H. Marker, and N. S. Key. Platelet microparticles are heterogeneous and highly dependent on the activation mechanism: Studies using a new digital flow cytometer. *Cytometry Part A*. 71(1):38–45, 2007. 10.1002/cyto.a.20354. 10.1002/cyto.a.20354 17216623

[22] Connor, D. E., T. Exner, D. D. F. Ma, and J. E. Joseph. The majority of circulating platelet-derived microparticles fail to bind annexin V, lack phospholipid-dependent procoagulant activity and demonstrate greater expression of glycoprotein Ib. *Thromb. Haemost.* 103(5):1044–1052, 2010. 10.1160/TH09-09-0644/ID/JR0644-6/BIB. 20390225 10.1160/TH09-09-0644

[23] Suades, R., T. Padró, and L. Badimon. The role of blood-borne microparticles in inflammation and hemostasis. *Semin. Thromb. Hemost.* 41(6):590–606, 2015. 10.1055/s-0035-1556591. 26276937 10.1055/s-0035-1556591

[24] Burnier, L., P. Fontana, B. R. Kwak, and A. S. Anne. Cell-derived microparticles in haemostasis and vascular medicine. *Thromb. Haemost.* 101(3):439–451, 2009. 10.1160/TH08-08-0521. 19277403

[25] Laffont, B., et al. Activated platelets can deliver mRNA regulatory Ago2 microRNA complexes to endothelial cells via microparticles. *Blood*. 122(2):253–261, 2013. 10.1182/blood-2013-03-492801. 23652806 10.1182/blood-2013-03-492801

[26] Lee, Y., S. El Andaloussi, and M. J. A. Wood. Exosomes and microvesicles: Extracellular vesicles for genetic information transfer and gene therapy. *Hum. Mol. Genet.* 2012. 10.1093/hmg/dds317. 10.1093/hmg/dds317 22872698

[27] Risitano, A. M., et al. The complement receptor 2/factor H fusion protein TT30 protects paroxysmal nocturnal hemoglobinuria erythrocytes from complement-mediated hemolysis and C3 fragment. *Blood*. 119(26):6307–6316, 2012. 10.1182/blood-2011-12-398792. 22577173 10.1182/blood-2011-12-398792

[28] Laffont, B., et al. Platelet microparticles reprogram macrophage gene expression and function. *Thromb. Haemost.* 115(2):311–323, 2016. 10.1160/TH15-05-0389/ID/JR0389-5/BIB. 26333874 10.1160/TH15-05-0389

[29] Owens, A. P., and N. MacKman. Microparticles in hemostasis and thrombosis. *Circulation Res*. 2011. 10.1161/CIRCRESAHA.110.233056. 10.1161/CIRCRESAHA.110.233056 PMC3144708 21566224

[30] Rand, M. L., et al. Phosphatidylserine exposure and other apoptotic-like events in Bernard-Soulier syndrome platelets. *Am. J. Hematol.* 85(8):584–592, 2010. 10.1002/ajh.21768. 20658588 10.1002/ajh.21768

[31] Li, X., and H. Cong. Platelet-derived microparticles and the potential of glycoprotein IIb/IIIa antagonists in treating acute coronary syndrome. *Tex Heart Inst J*. 2(36):134–139, 2009. PMC2676586 19436807

[32] Gemmell, C. H., M. V. Sefton, and E. L. Yeo. Communication platelet-derived microparticle formation involves glycoprotein IIb-IIIa. *J. Biol. Chem.* 268(20):14586–14589, 1993. 8325838

[33] Lazar, S., and L. E. Goldfinger. Platelet microparticles and miRNA transfer in cancer progression: many targets, modes of action, and effects across cancer stages. *Frontiers Media S.A.* 2018. 10.3389/fcvm.2018.00013. 10.3389/fcvm.2018.00013 PMC5850852 29564336

[34] Lazar, S., and L. E. Goldfinger. Platelets and extracellular vesicles and their cross talk with cancer. *Blood*. 137(23):2125–2132, 2021. 10.1182/BLOOD.2019000962. 33940593 10.1182/blood.2019004119 PMC8351904

[35] Szilágyi, B., et al. Platelet microparticles enriched in miR-223 reduce ICAM-1-dependent vascular inflammation in septic conditions. *Front. Physiol.* 2021. 10.3389/fphys.2021.658524. 10.3389/fphys.2021.658524 PMC8201999 34135769

[36] Żmigrodzka, M., O. Witkowska-Piłaszewicz, and A. Winnicka. Platelets extracellular vesicles as regulators of cancer progression—an updated perspective. *Int. J. Mol. Sci.* 21(15):1–18, 2020. 10.3390/ijms21155195. 10.3390/ijms21155195 PMC7432409 32707975

[37] Voudoukis, E., et al. Distinct features of circulating microparticles and their relationship with disease activity in inflammatory bowel disease. *Ann. Gastroenterol.* 29(2):180–187, 2016. 10.20524/aog.2016.0010. 27065731 10.20524/aog.2016.0010 PMC4805738

[38] Cloutier, N., R. W. Farndale, A. Brisson, and E. Boilard. Functions of platelet microparticles in inflammatory arthritis. *Blood.* 118(21):35, 2011. 10.1182/blood.v118.21.sci-35.sci-35.

[39] Boilard, E., et al. Platelets amplify inflammation in arthritis via collagen-dependent microparticle production. *Science*. 327:2010, 1979. 10.1126/science.1181928. 10.1126/science.1181928 PMC2927861 20110505

[40] Guo, M., et al. Platelet-derived microRNA-223 attenuates TNF-α induced monocytes adhesion to arterial endothelium by targeting ICAM-1 in Kawasaki disease”. *Front. Immunol.* 2022. 10.3389/fimmu.2022.922868. 10.3389/fimmu.2022.922868 PMC9379370 35983051

[41] Kyselova, A., et al. Platelet-derived calpain cleaves the endothelial protease-activated receptor 1 to induce vascular inflammation in diabetes. *Basic Res Cardiol*. 2020. 10.1007/s00395-020-00833-9. 10.1007/s00395-020-00833-9 PMC7716944 33258989

[42] Rautou, P. E., et al. Microparticles from human atherosclerotic plaques promote endothelial ICAM-1-dependent monocyte adhesion and transendothelial migration. *Circ. Res.* 108(3):335–343, 2011. 10.1161/CIRCRESAHA.110.237420. 21164106 10.1161/CIRCRESAHA.110.237420

[43] Kassassir, H., I. Papiewska-Pająk, J. Kryczka, J. Boncela, and M. A. Kowalska. Platelet-derived microparticles stimulate the invasiveness of colorectal cancer cells via the p38MAPK-MMP-2/MMP-9 axis. *Cell Communication Signaling*. 2023. 10.1186/s12964-023-01066-8. 10.1186/s12964-023-01066-8 PMC9990213 36882818

[44] Signorelli, S. S., et al. Inter-relationship between platelet-derived microparticles and oxidative stress in patients with venous thromboembolism. *Antioxidants*. 9(12):1–14, 2020. 10.3390/antiox9121217. 10.3390/antiox9121217 PMC7761576 33276677

[45] Zahran, A., S. Sayed, H. A. Abd El Hafeez, W. A. Khalifa, N. Mohamed, and H. Hetta. Circulating microparticle subpopulation in metabolic syndrome: relation to oxidative stress and coagulation markers. *Diabetes Metab. Syndr. Obes.* 12:485–493, 2019. 10.2147/dmso.s191750. 31043798 10.2147/DMSO.S191750 PMC6469468

[46] Hayon, Y., O. Dashevsky, E. Shai, D. Varon, and R. R. Leker. Platelet microparticles promote neural stem cell proliferation, survival and differentiation. *J Mol Neurosci*. 47(3):659–665, 2012. 10.1007/s12031-012-9711-y. 22290563 10.1007/s12031-012-9711-y

[47] Brill, A., O. Dashevsky, J. Rivo, Y. Gozal, and D. Varon. Platelet-derived microparticles induce angiogenesis and stimulate post-ischemic revascularization. *Cardiovasc. Res.* 67(1):30–38, 2005. 10.1016/j.cardiores.2005.04.007. 15878159 10.1016/j.cardiores.2005.04.007

[48] Li, X., and Q. Wang. Platelet-derived microparticles and autoimmune diseases. *Int J Mol Sci*. 2023. 10.3390/ijms241210275. 10.3390/ijms241210275 PMC10298940 37373420

[49] Boudreau, L. H., et al. Platelets release mitochondria serving as substrate for bactericidal group IIA-secreted phospholipase a to promote inflammation. *Blood*. 124(14):2173–2183, 2014. 10.1182/blood-2014-05-573543. 25082876 10.1182/blood-2014-05-573543 PMC4260364

[50] Joop, K., et al. Microparticles from patients with multiple organ dysfunction syndrome and sepsis support coagulation through multiple mechanisms. *Thromb. Haemost.* 85(5):810–820, 2001. 10.1055/S-0037-1615753. 11372673

[51] Mooberry, M. J., R. Bradford, E. L. Hobl, F. C. Lin, B. Jilma, and N. S. Key. Procoagulant microparticles promote coagulation in a factor XI-dependent manner in human endotoxemia. *J Thromb Haemost*. 14(5):1031–1042, 2016. 10.1111/JTH.13285. 26857798 10.1111/jth.13285 PMC4870121

[52] Bucciarelli, P., et al. Circulating microparticles and risk of venous thromboembolism. *Thromb. Res.* 129(5):591–597, 2012. 10.1016/J.THROMRES.2011.08.020. 21908018 10.1016/j.thromres.2011.08.020

[53] Nomura, S., et al. Platelet-derived microparticles may influence the development of atherosclerosis in diabetes mellitus. *Atherosclerosis*. 116(2):235–240, 1995. 10.1016/0021-9150(95)05551-7. 7575778 10.1016/0021-9150(95)05551-7

[54] Melki, I., N. Tessandier, A. Zufferey, and E. Boilard. Platelet microvesicles in health and disease. *Platelets*. 28(3):214–221, 2017. 10.1080/09537104.2016.1265924. 28102737 10.1080/09537104.2016.1265924

[55] Yadav, P., A. R. Panigrahi, S. K. Beura, and S. K. Singh. Platelet-derived microvesicles induce intracellular calcium mobilization in human platelets. *Cell Biol. Int.* 47(12):1964–1975, 2023. 10.1002/cbin.12084. 37650361 10.1002/cbin.12084

[56] Berckmans, R. J., R. Nieuwland, A. N. Böing, F. P. H. T. M. Romijn, C. E. Hack, and A. Sturk. Cell-derived microparticles circulate in healthy humans and support low grade thrombin generation. *Thromb. Haemost.* 85(4):639–646, 2001. 10.1055/s-0037-1615646. 11341498

[57] Shah, M. D., A. L. Bergeron, J. F. Dong, and J. A. Lòpez. Flow cytometric measurement of microparticles: Pitfalls and protocol modifications. *Platelets*. 19:365–372, 2008. 10.1080/09537100802054107. 18791943 10.1080/09537100802054107

[58] Schmitz, G., et al. European working group on clinical cell analysis: consensus protocol for the flow cytometric characterisation of platelet function. *Thromb. Haemost.* 79(5):885–896, 1998. 9609215

[59] Roka-Moiia, Y., et al. Shear-mediated platelet activation in the free flow II: Evolving mechanobiological mechanisms reveal an identifiable signature of activation and a bi-directional platelet dyscrasia with thrombotic and bleeding features. *J. Biomech.* 2021. 10.1016/j.jbiomech.2021.110415. 10.1016/j.jbiomech.2021.110415 PMC8513575 34052772

[60] Araki, R., et al. A characteristic flow cytometric pattern with broad forward scatter and narrowed side scatter helps diagnose immune thrombocytopenia (ITP). *Int. J. Hematol.* 108(2):151–160, 2018. 10.1007/s12185-018-2454-y. 29663189 10.1007/s12185-018-2454-y

[61] Scavone, M., S. Bozzi, T. Mencarini, G. Podda, M. Cattaneo, and A. Redaelli. Platelet adhesion and thrombus formation in microchannels: The effect of assay-dependent variables. *Int. J. Mol. Sci.* 21(3):1–11, 2020. 10.3390/ijms21030750. 10.3390/ijms21030750 PMC7037340 31979370

[62] Neeves, K. B., et al. Sources of variability in platelet accumulation on type 1 fibrillar collagen in microfluidic flow assays. *PLoS One*. 2013. 10.1371/journal.pone.0054680. 10.1371/journal.pone.0054680 PMC3552855 23355889

[63] Colace, T., E. Falls, X. L. Zheng, and S. L. Diamond. Analysis of morphology of platelet aggregates formed on collagen under laminar blood flow. *Ann. Biomed. Eng.* 39(2):922–929, 2011. 10.1007/S10439-010-0182-4. 20949319 10.1007/s10439-010-0182-4

[64] Suades, R., T. Padró, G. Vilahur, and L. Badimon. Circulating and platelet-derived microparticles in human blood enhance thrombosis on atherosclerotic plaques. *Thromb. Haemost.* 108(6):1208–1219, 2012. 10.1160/TH12-07-0486/ID/JR0486-7/BIB. 23138460 10.1160/TH12-07-0486

[65] Zaidi, T. N., L. V. McIntire, D. H. Farrell, and P. Thiagarajan. Adhesion of platelets to surface-bound fibrinogen under flow. *Blood*. 88(8):2967–2972, 1996. 10.1182/blood.v88.8.2967.bloodjournal8882967. 8874193

[66] Savage, B., E. Saldívar, and Z. M. Ruggeri. Initiation of platelet adhesion by arrest onto fibrinogen or translocation on von Willebrand factor. *Cell*. 84:289–297, 1996. 8565074 10.1016/s0092-8674(00)80983-6

[67] M. B. Horev *et al.* Differential dynamics of early stages of platelet adhesion and spreading on collagen IV- and fibrinogen-coated surfaces. *F1000Research. *9 449 2020. 10.12688/f1000research.23598.2. 10.12688/f1000research.23598.1 PMC7281675 32566134

[68] Bonnefoy, A., Q. Liu, C. Legrand, and M. M. Frojmovic. Efficiency of Platelet Adhesion to Fibrinogen Depends on both Cell Activation and Flow. *Biophys. J.* 78(6):2834–2843, 2000. 10.1016/S0006-3495(00)76826-3. 10827966 10.1016/S0006-3495(00)76826-3 PMC1300871

[69] Thornber, K., O. J. T. McCarty, S. P. Watson, and C. J. Pears. Distinct but critical roles for integrin alphaIIbbeta3 in platelet lamellipodia formation on fibrinogen, collagen-related peptide and thrombin. *FEBS J.* 273(22):5032–5043, 2006. 10.1111/J.1742-4658.2006.05500.X. 17032352 10.1111/j.1742-4658.2006.05500.x

[70] Beumer, S., M. J. W. Ijsseldijk, P. G. De Groot, and J. J. Sixma. Platelet adhesion to fibronectin in flow: Dependence on surface concentration and shear rate, role of platelet membrane glycoproteins GP IIb/IIIa and VLA-5, and inhibition by heparin. *Blood*. 84(11):3724–3733, 1994. 10.1182/blood.v84.11.3724.bloodjournal84113724. 7949128

[71] Rodenberg, E. J., and F. M. Pavalko. Peptides derived from fibronectin type III connecting segments promote endothelial cell adhesion but not platelet adhesion: Implications in tissue-engineered vascular grafts. *Tissue Eng.* 13(11):2653–2666, 2007. 10.1089/ten.2007.0037. 17883325 10.1089/ten.2007.0037

[72] McCarty, O. J. T., et al. Evaluation of the role of platelet integrins in fibronectin-dependent spreading and adhesion. *Journal of Thrombosis and Haemostasis*. 2(10):1823–1833, 2004. 10.1111/j.1538-7836.2004.00925.x. 15456495 10.1111/j.1538-7836.2004.00925.x

[73] Maurer, E., et al. Fibrillar cellular fibronectin supports efficient platelet aggregation and procoagulant activity. *Thromb. Haemost.* 114(6):1175–1188, 2015. 10.1160/TH14-11-0958. 26245230 10.1160/TH14-11-0958

[74] Bastida, E., G. Escolar, A. Ordinas, and J. J. Sixma. Fibronectin is required for platelet adhesion and for thrombus formation on subendothelium and collagen surfaces. *Blood*. 70(5):1437–1442, 1987. 10.1182/BLOOD.V70.5.1437.1437. 3663940

[75] Cho, J., and D. F. Mosher. Role of fibronectin assembly in platelet thrombus formation. *J Thromb Haemost*. 4(7):1461–1469, 2006. 10.1111/J.1538-7836.2006.01943.X. 16839338 10.1111/j.1538-7836.2006.01943.x

[76] Hindriks, G., M. J. W. Ijsseldijk, A. Sonnenberg, J. J. Sixma, and P. G. De Groot. Platelet adhesion to laminin: role of Ca2+ and Mg2+ ions, shear rate, and platelet membrane glycoproteins. *Blood.* 79(4):928–935, 1992. 10.1182/BLOOD.V79.4.928.928. 1737101

[77] Inoue, O., K. Suzuki-Inoue, and Y. Ozaki. Redundant mechanism of platelet adhesion to laminin and collagen under flow: Involvement of von Willebrand factor and glycoprotein Ib-IX-V. *J Biol Chem*. 283(24):16279–16282, 2008. 10.1074/jbc.C700241200. 18450753 10.1074/jbc.C700241200

[78] Inoue, O., et al. Laminin stimulates spreading of platelets through integrin alpha6beta1-dependent activation of GPVI. *Blood*. 107(4):1405–1412, 2006. 10.1182/BLOOD-2005-06-2406. 16219796 10.1182/blood-2005-06-2406 PMC1895394

[79] Ill, C. R., E. Engvall, and E. Ruoslahti. Adhesion of platelets to laminin in the absence of activation. *J Cell Biol*. 99(6):2140–2145, 1984. 10.1083/jcb.99.6.2140. 6501416 10.1083/jcb.99.6.2140 PMC2113577

[80] Varga-Szabo, D., I. Pleines, and B. Nieswandt. Cell adhesion mechanisms in platelets. *Arterioscler. Thromb. Vasc. Biol.* 28(3):403–413, 2008. 10.1161/ATVBAHA.107.150474. 18174460 10.1161/ATVBAHA.107.150474

[81] Roka-Moiia, Y., et al. Platelet activation via shear stress exposure induces a differing pattern of biomarkers of activation versus biochemical agonists. *Thromb. Haemost.* 120(5):776–792, 2020. 10.1055/s-0040-1709524. 32369849 10.1055/s-0040-1709524 PMC8577487

[82] Merten, M., R. Pakala, P. Thiagarajan, and C. R. Benedict. Platelet microparticles promote platelet interaction with subendothelial matrix in a glycoprotein IIb/IIIa-dependent mechanism. *Circulation*. 99(19):2577–2582, 1999. 10.1161/01.CIR.99.19.2577. 10330391 10.1161/01.cir.99.19.2577

[83] Holme, P. A., N. O. Solum, F. Brosstad, T. Pedersen, and M. Kveine. Microvesicles bind soluble fibrinogen, adhere to immobilized fibrinogen and coaggregate with platelets. *Thromb. Haemost.* 79(2):389–394, 1998. 10.1055/s-0037-1614997. 9493596

[84] Siljander, P., O. Carpen, and R. Lassila. Platelet-derived microparticles associate with fibrin during thrombosis. *Blood*. 87(11):4651–4663, 1996. 10.1182/blood.v87.11.4651.bloodjournal87114651. 8639834

[85] Artemenko, E. O., S. I. Obydennyi, K. S. Troyanova, G. A. Novichkova, D. Y. Nechipurenko, and M. A. Panteleev. Adhesive properties of plasma-circulating and platelet-derived microvesicles from healthy individuals. *Thromb. Res.* 233:119–126, 2024. 10.1016/j.thromres.2023.11.018. 38039724 10.1016/j.thromres.2023.11.018

[86] Neeves, K. B., O. J. T. Mccarty, A. J. Reininger, M. Sugimoto, and M. R. King. Flow-dependent thrombin and fibrin generation in vitro: opportunities for standardization: communication from SSC of the ISTH. *Journal of Thrombosis and Haemostasis*. 12(3):418–420, 2014. 10.1111/jth.12482. 24330648 10.1111/jth.12482

[87] Puddu, P., G. M. Puddu, E. Cravero, S. Muscari, and A. Muscari. The involvement of circulating microparticles in inflammation, coagulation and cardiovascular diseases. *Can. J. Cardiol.* 2010. 10.1016/S0828-282X(10)70371-8. 10.1016/s0828-282x(10)70371-8 PMC2886541 20386775

[88] Guo, J., et al. Platelet-derived microparticles and their cargos: The past, present and future. *Asian J Pharm Sci*. 2024. 10.1016/j.ajps.2024.100907. 10.1016/j.ajps.2024.100907 PMC11016590 38623487

[89] Puhm, F., E. Boilard, and K. R. MacHlus. Platelet extracellular vesicles; beyond the blood. *Arterioscler. Thromb. Vasc. Biol.* 41(1):87–96, 2021. 10.1161/ATVBAHA.120.314644. 33028092 10.1161/ATVBAHA.120.314644 PMC7769913

[90] Somajo, S., R. L. Koshiar, E. Norström, and B. Dahlbäck. Protein S and factor V in regulation of coagulation on platelet microparticles by activated protein C. *Thromb. Res.* 134(1):144–152, 2014. 10.1016/J.THROMRES.2014.04.031. 24835672 10.1016/j.thromres.2014.04.031

[91] Tans, G., J. Rosing, M. Thomassen, M. Heeb, R. Zwaal, and J. Griffin. Comparison of Anticoagulant and Procoagulant Activities of Stimulated Platelets and Platelet-Derived Microparticles. *Blood*. 77(12):2641–2648, 1991. 10.1182/BLOOD.V77.12.2641.2641. 2043766

[92] Boilard, E., A. C. Duchez, and A. Brisson. The diversity of platelet microparticles. *Curr. Opin. Hematol.* 22(5):437–444, 2015. 10.1097/MOH.0000000000000166. 26214207 10.1097/MOH.0000000000000166

