# Supplementary material for: The Role of Platelet-Derived Microparticles in Platelet Adhesion and Aggregation to Immobilized Extracellular Matrix Proteins Under Flow
Source: Cell Mol Bioeng. 2026 Jun 10;19(3):387–405. doi: 10.1007/s12195-026-00920-2 (PMC13365112; doi:10.1007/s12195-026-00920-2)
Supplement: Supplementary file 1 — Supplementary file1 (DOCX 204 kb) [file 12195_2026_920_MOESM1_ESM.docx]

**Supplementary material**


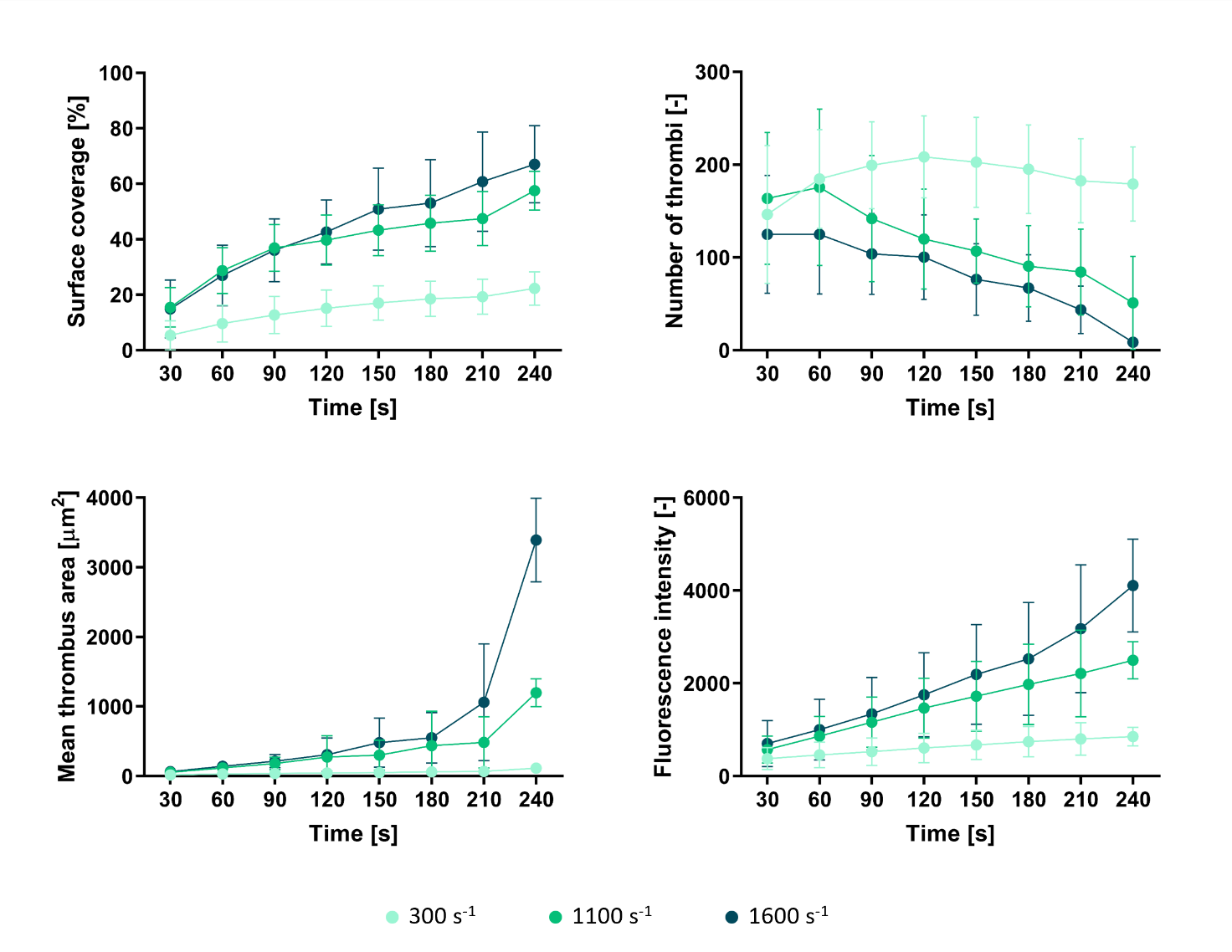


Figure S1. Thrombus formation kinetics during the perfusion, at three shear rates, of healthy whole blood through a 100 µg/mL collagen-coated microchannel for 4 minutes. Thrombus formation is expressed as surface coverage, number of thrombi, mean thrombus area, and fluorescence intensity, computed from fluorescence images acquired every 30 seconds at a fixed location along the coated channel. N = 12. Data are reported as mean ± standard deviation.
